# Using Reports of Symptoms and Diagnoses on Social Media to Predict COVID-19 Case Counts in Mainland China: Observational Infoveillance Study

**DOI:** 10.2196/19421

**Published:** 2020-05-28

**Authors:** Cuihua Shen, Anfan Chen, Chen Luo, Jingwen Zhang, Bo Feng, Wang Liao

**Affiliations:** 1 Department of Communication University of California, Davis Davis, CA United States; 2 Department of Science Communication and Science Policy University of Science and Technology of China Hefei China; 3 School of Journalism and Communication Tsinghua University Beijing China; 4 Department of Public Health Sciences University of California, Davis Davis, CA United States

**Keywords:** COVID-19, SARS-CoV-2, novel coronavirus, infectious disease, social media, Weibo, China, disease surveillance, surveillance, infoveillance, infodemiology

## Abstract

**Background:**

Coronavirus disease (COVID-19) has affected more than 200 countries and territories worldwide. This disease poses an extraordinary challenge for public health systems because screening and surveillance capacity is often severely limited, especially during the beginning of the outbreak; this can fuel the outbreak, as many patients can unknowingly infect other people.

**Objective:**

The aim of this study was to collect and analyze posts related to COVID-19 on Weibo, a popular Twitter-like social media site in China. To our knowledge, this infoveillance study employs the largest, most comprehensive, and most fine-grained social media data to date to predict COVID-19 case counts in mainland China.

**Methods:**

We built a Weibo user pool of 250 million people, approximately half the entire monthly active Weibo user population. Using a comprehensive list of 167 keywords, we retrieved and analyzed around 15 million COVID-19–related posts from our user pool from November 1, 2019 to March 31, 2020. We developed a machine learning classifier to identify “sick posts,” in which users report their own or other people’s symptoms and diagnoses related to COVID-19. Using officially reported case counts as the outcome, we then estimated the Granger causality of sick posts and other COVID-19 posts on daily case counts. For a subset of geotagged posts (3.10% of all retrieved posts), we also ran separate predictive models for Hubei province, the epicenter of the initial outbreak, and the rest of mainland China.

**Results:**

We found that reports of symptoms and diagnosis of COVID-19 significantly predicted daily case counts up to 14 days ahead of official statistics, whereas other COVID-19 posts did not have similar predictive power. For the subset of geotagged posts, we found that the predictive pattern held true for both Hubei province and the rest of mainland China regardless of the unequal distribution of health care resources and the outbreak timeline.

**Conclusions:**

Public social media data can be usefully harnessed to predict infection cases and inform timely responses. Researchers and disease control agencies should pay close attention to the social media infosphere regarding COVID-19. In addition to monitoring overall search and posting activities, leveraging machine learning approaches and theoretical understanding of information sharing behaviors is a promising approach to identify true disease signals and improve the effectiveness of infoveillance.

## Introduction

Since the outbreak of coronavirus disease (COVID-19) in December 2019 in Wuhan, Hubei Province, China [1,2], the novel coronavirus has affected more than 200 countries and territories worldwide. As of May 16, 2020, there were more than 4 million confirmed cases of COVID-19 and over 300,000 deaths [3]. Amid many unknown factors, severe lack of laboratory testing capacity, delays in case reports, variations in local COVID-19 responses, and uncoordinated communication pose tremendous challenges for monitoring the dynamics of the epidemic and developing policies and targeted interventions for resource allocation. 

When conventional disease surveillance capacity is limited, publicly available social media and internet data can play a crucial role in uncovering the hidden dynamics of an emerging outbreak [4]. Research in digital disease surveillance, also referred to as infoveillance or infodemiology, has shown great promise in the useful employment of internet data to track the real time development of public attention, sentiment, and health [5-8]. Specifically, data based on internet searches and social media activities can nowcast and forecast disease prevalence as a supplement to conventional surveillance methods for various infectious diseases [5-7,9-14].

One of the best-known examples of digital disease surveillance is Google Flu Trends, which used real time Google search terms to predict clinical incidence rates of influenza with great initial success [13,14]. Data from social media platforms such as Twitter have also been shown to be effective in predicting and tracking various epidemics, such as influenza [10,12] and Zika virus [15], with varying degrees of success. However, digital surveillance data present unique challenges. For example, after its release in 2008, Google Flu Trends became less accurate over time, consistently overestimating flu prevalence during 2011-2013. The prediction error was partially attributed to people’s changing search behaviors as well as increased public attention to the epidemic itself, which fueled awareness-related search queries that were not strongly related to disease incidence [7,16]. Compared to aggregated search queries, user-generated social media data have the advantage of being more direct and granular, allowing researchers to mine specific content to reflect actual illness. However, media attention to emerging diseases can fuel social media activities, resulting in a deluge of discussions that dilute true disease signals of actual infection cases; thus, predictions are less accurate [12].

The unprecedented magnitude and transmission speed of COVID-19 brought about massive social media activities as people isolated themselves in their homes to break the infection chain [17]. Massive social media data inevitably contain massive noise (eg, public reactions and awareness of the disease), which can be counterproductive for disease forecasting. A few early infoveillance studies tracked public discussion of COVID-19 and patient characteristics on Weibo, the most popular public social media site in China [18-21]. Two studies suggested that COVID-19–related Weibo posts and search queries can be used to predict disease prevalence [19,22]. However, these studies relied upon coarse-grained social media data and query data based on a few keywords with a short time window at the onset of the outbreak [19,22]. As such, the predictive accuracy and result interpretability of these studies are limited by the same pitfalls of infoveillance studies mentioned above. There are many reasons to search for and discuss COVID-19 on social media, especially because the disease has received substantial media coverage and many countries are under mandatory lockdown. To more accurately predict infection cases and inform a rapid response, it is therefore critical to use granular and specific social media data to identify reliable disease signals (ie, “sick posts” reporting symptoms and diagnosis).

Here, we present an infoveillance effort to collect and analyze COVID-19–related posts on Weibo and to identify specific types of Weibo posts that can predict COVID-19 case counts in mainland China. To our knowledge, this study involves the largest, most comprehensive, and most granular collection of social media data related to COVID-19 in the Chinese language, far exceeding the scale, granularity, and timespan of similar studies [19,22]. We built a Weibo user pool of 250 million people, approximately half the active Weibo user population [23]. Using a comprehensive list of 167 keywords associated with COVID-19, we retrieved around 15 million social media posts from November 1, 2019 to March 31, 2020. With greatly increased data granularity, we developed a supervised machine learning classifier to distinguish “sick posts,” which are reports of one’s own and other people’s symptoms or diagnosis, from other COVID-19 related posts that could dilute disease signals from the data stream. Using the officially reported case counts as the outcome, we compared the predictive power of sick posts versus other COVID-19 posts. We show evidence that sick posts predicted the daily cases reported by the Chinese Center for Disease Control and Prevention (China CDC) up to 14 days in advance, while other COVID-19–related posts had much weaker predictive power. For the subset of geotagged posts, we found that the predictive pattern held true for both Hubei province and the rest of mainland China. Our work demonstrates a viable method to identify disease signals through reports of symptoms or diagnosis rather than relying upon general discussion of COVID-19, making a significant contribution to the infoveillance literature. 

## Methods

### Data Collection

The social media data used in this study were collected from a popular Chinese microblog platform, Weibo, which had over 516 million monthly active users at the end of 2019 [23]. Weibo is very similar to Twitter, access to which is blocked in mainland China. Unlike Twitter, Weibo does not provide large-scale public application programming interface (API) access to its database. Weibo enables keyword-based advanced searching of posts via its web interface; however, per Weibo policy, the output of these searches is limited to 50 pages (or around 1000 posts). Therefore, large-scale public data access is notoriously difficult.

**Figure 1:**
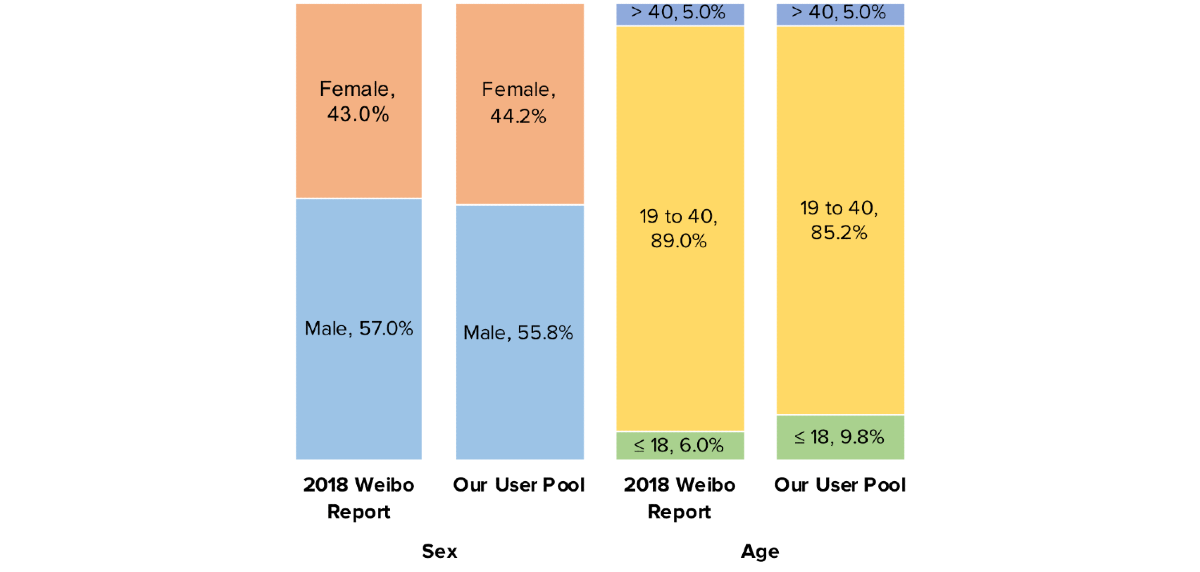
Demographic composition of our Weibo user pool compared to that in the 2018 Annual Sina Weibo user report. Age is reported in years.

To bypass these limitations, we employed a Weibo user pool originally built in 2018, which started from 5 million active Weibo users obtained in our previous research unrelated to COVID-19 [24,25]. We then retrieved the initial 5 million users’ followers and followees (second degree users), the followers and followees of the second degree users (third degree users), etc., until no new users were found. This snowball process resulted in a pool of 250 million users (with bots filtered out), which represents approximately 48.4% of all monthly active Weibo users in 2019 [23] and is similar to the 2018 population of Weibo users in terms of self-reported sex and age distribution [26] (see Figure 1). 

### COVID-19 Posts

Following best practices for content retrieval and analysis [27], we generated a comprehensive list of keywords related to COVID-19 through close observation of Weibo posts every day from late January to March 2020. We then retrieved COVID-19 posts by searching all posts by users in the user pool with 167 keywords covering general terms related to the epidemic, such as coronavirus and pneumonia, as well as specific locations (eg, “Wuhan”), drugs (eg, “remdesivir”) and preventive measures (eg, “mask”). For a complete keyword list, see Multimedia Appendix 1, Table A).

After removing duplicates (ie, reposts of original posts), we retained 14,983,647 posts sent between November 1, 2019 (ie, 30 days before the first confirmed cases) and March 31, 2020 (to access the Weibo dataset on COVID-19, see [28]).

A subset of 464,111/14,983,647 of these posts (3.10%) were tagged with geographic information. We distinguished between posts sent within Hubei province (ie, the epicenter; 169,340/14,983,647; 36.49%) and those from elsewhere in mainland China (294,771/14,983,647; 63.51%).

### Sick Posts

We conceptually defined “sick posts” as posts that report any symptoms or diagnoses that are likely related to COVID-19 based on published research and news reports from the medical social media site DXY.cn [29]. We collected a broad list of symptoms, including common symptoms such as cough and shortness of breath and uncommon symptoms such as diarrhea. Sick posts can be further categorized into “ingroup sick posts,” which we defined as posts that disclose the user’s own or immediate family members’ symptoms or diagnoses, and “outgroup sick posts,” which report symptoms and diagnoses of people not in the user’s immediate family. The reason for the a priori categorization is that people tend to have firsthand and more accurate information about their own or immediate family members’ medical conditions; meanwhile, they have much less reliable information about people outside of their household, especially during a national lockdown. All posts that were obtained using the 167 keywords but did not fall into these categories were classified as “other COVID-19 posts.” We provide an example of an ingroup sick post below (translated and edited for brevity): 

During the SARS epidemic in 2003, I got pneumonia with symptoms of fever and cough, was suspected of being infected with SARS, and ended up being hospitalized for more than a month. Now we got COVID-19 in 2020 and I started coughing again, which has lasted for more than a month. What a mess <Face Palm> (Posted 10:23 PM, January 29, 2020)

We also provide an example of an outgroup sick post:

One man in another village drank too much. He said he felt sick and had cold symptoms. His brother measured his temperature which turned out to be 38 Celsius. His brother called 120 and sent him to hospital. The whole village was shocked and everyone was afraid to go outside. (Posted 10:14 PM, January 29, 2020)

We used supervised machine learning algorithms to identify sick posts from the keyword-retrieved COVID-19 posts. We first sampled 11,575 posts in proportion to the retrieved posts across 5 months of data collection. Next, 11 human judges annotated whether a post was an ingroup sick post, outgroup sick post, or other COVID-19 post. The judges independently annotated a subset of 138 posts and achieved high agreement (Krippendorff α=.945) before they divided and annotated the remaining posts. Then, the annotated posts were used to train machine learning models with various algorithms. Based on the classification performance (see Table 1), we selected the model using the random forest algorithm (F1 score=0.880). The model classified the 14,983,647 COVID-19 posts into 394,658 (2.63%) ingroup sick posts, 97,635 (0.65%) outgroup sick posts, and 14,491,354 (96.71%) other COVID-19 posts. Because of the low number of outgroup sick posts, we combined ingroup and outgroup sick posts in subsequent analyses. 

**Figure 2:**
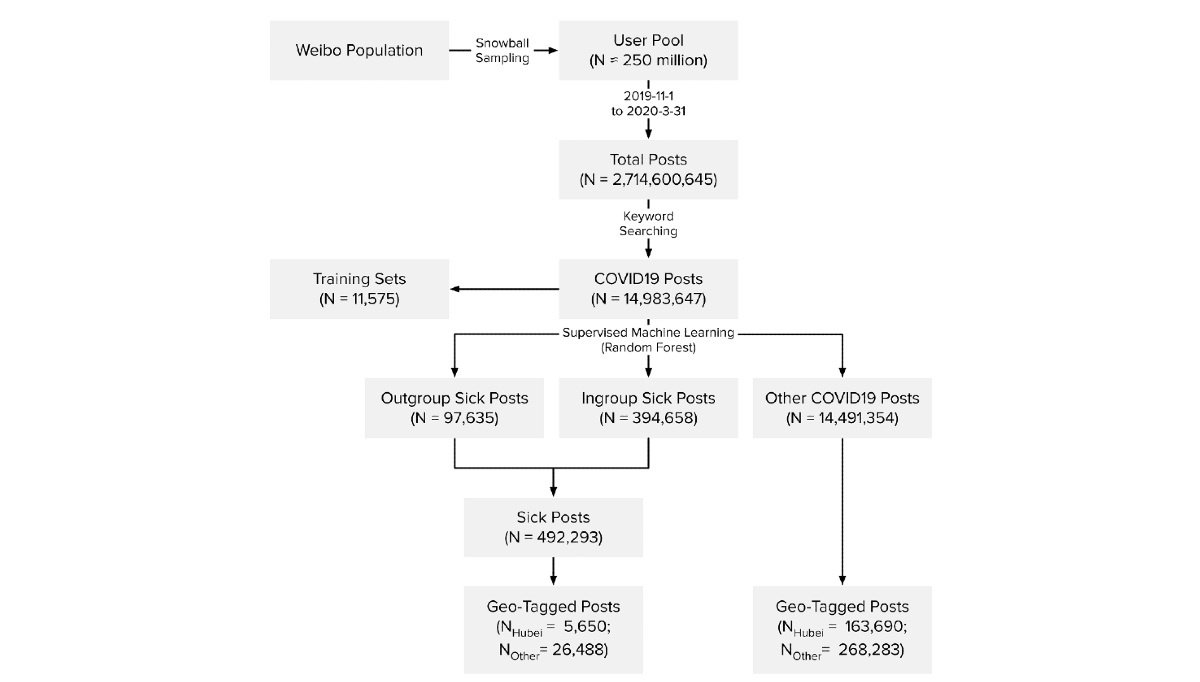
Weibo data collection and classification procedure.

Among the subset of geotagged COVID-19 posts (464,111/14,983,647, 3.10% of all retrieved posts), 5,650 sick posts (1.2%) and 163,690 other COVID-19 posts (35.3%) were tagged in Hubei; meanwhile, 26,488 sick posts (5.7%) and 268,283 other COVID-19 posts (57.8%) were from elsewhere in mainland China. These post counts were then aggregated by days. To control for the day-to-day fluctuations of Weibo posts, we further normalized these numbers against the daily counts of all Weibo posts generated by our user pool. The normalized sick post and other COVID-19 post counts can be interpreted as counts per 1 million posts. Figure 2 summarizes our data collection and classification process.

**Table 1:** Performance of machine learning models in classifying sick posts.

Model	F1 score	Precision	Accuracy	Recall
Decision tree	0.835	0.840	0.830	0.830
Extra tree	0.785	0.785	0.785	0.785
Extra trees	0.878	0.881	0.885	0.885
K nearest neighbors	0.810	0.819	0.819	0.819
Multilayer perceptron	0.847	0.845	0.851	0.851
Support vector machine	0.877	0.877	0.878	0.878
Random forest	0.880	0.885	0.888	0.888

### COVID-19 Daily Case Counts

**Figure 3:**
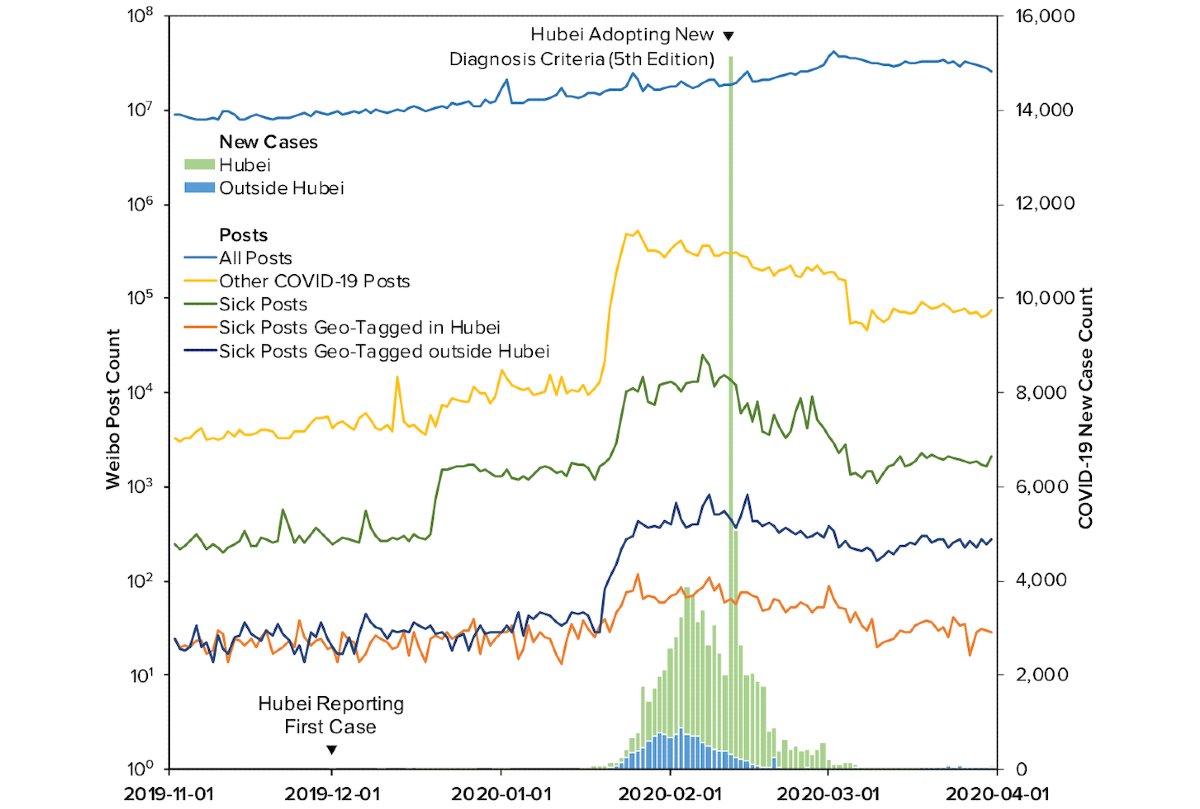
Daily Weibo posts and confirmed COVID-19 cases between November 1, 2019 and March 31, 2020.

We collected the daily new case counts in mainland China from China CDC on May 8, 2020. China CDC’s official website started collating data on January 16, 2020. Earlier counts were obtained from Huang et al [1] and validated against relevant briefings from the National Health Commission. The final case data cover the same period from November 1, 2019, to March 31, 2020, within which the first reported COVID-19 clinical case dates back to December 1, 2019. We also distinguished between cases within and outside Hubei (see Figure 3).

It is noteworthy that China CDC released seven editions of diagnostic criteria throughout the time period covered in this study and thus introduced systematic changes to the case counts. Particularly, on February 12, 2020, Hubei province started to implement the fifth edition of the COVID-19 diagnostic criteria released on February 4, 2020. This led to a temporary surge of new cases [30]. The impact of this incident was controlled for in our analyses, as discussed in the section below. After close comparison of each edition, we concluded that the changes among other editions of the diagnostic criteria were relatively minor, and their release dates did not appear to be associated with abrupt changes in the case counts; therefore, we did not further control for them.

### Statistical Analysis

We performed Granger causality tests [31] to discover if an increase of sick posts forecasted an increase of new cases, as formulated in the following linear model:


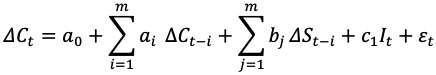


where *Ct* is the difference in new case counts at day *t* from day *t –*1, *S_t-i_* is the difference in sick post counts (normalized) at day *t* from day *t –*1, and *I_t_* is a time-varying binary variable that equals 1 on February 12, 2020, the day on which Hubei adopted the fifth edition of the diagnostic criteria. This binary variable controls for the exogenous pulse of case counts [32]. Since we collected Weibo posts from as early as November 1, 2019, 30 days before the first reported case of COVID-19 on December 1, 2019, we were able to test up to 29 lags of such posts (ie, *m* ≤ 29). The model is further explained as follows.

First, difference scores instead of raw new case counts were used because Dickey-Fuller tests for the raw counts could not reject nonstationarity (ie, the presence of a unit root) for lag 3–29 at a 5% confidence level (see Table B in Multimedia Appendix 1). Both stationarity and the inclusion of autoregressive terms are required by Granger causality. In contrast, the Dicky-Fuller tests suggested that the difference scores of the case counts were stationary: nonstationarity was rejected for lag 1–12 at a 1% confidence level and for lag 13–29 at a 5% confidence level (see Table B in Multimedia Appendix 1). The Dickey-Fuller tests reached the same conclusion for the stationarities of the sick post counts and their difference scores (see Table B in Multimedia Appendix 1). We thus also used the difference scores instead of the raw counts to reduce correlations among lag terms of sick post counts. This more clearly identifies their independent effects on case counts. In short, these difference scores can be interpreted as “daily-additional” cases or Weibo posts in addition to the counts from the previous day.  

Second, to determine the number of lag terms to include (ie, *m* in the above formula), we compared model fit statistics while iteratively adding lag terms. The model comparison suggested that the inclusion of more lags continuously improved the model fit up to the maximum lags (ie, 29; see Table C in Multimedia Appendix 1). However, the parameter estimates did not change qualitatively after including more than 20 lags (see Tables D and E in Multimedia Appendix 1). For parsimony and statistical power, we settled at 20 lags for the following analyses. 

Finally, we included a binary variable to control for the change in the diagnostic criteria of COVID-19 on Feb 12, 2020, following the procedure of intervention analysis [33]. Because this change is unlikely to induce permanent changes to case counts, an instant pulse function was applied at the date of the change. We also tested models that allowed the effect to linearly decay in 2, 3, 4, or 5 days; these models fitted the data more poorly than the model with an instant pulse (see Table F in Multimedia Appendix 1).

## Results

Ordinary least squares regression with robust standard errors was used to estimate the final models. With 20 lag terms in the model, the modeled data include daily-additional new COVID-19 cases from December 1, 2019 to March 31, 2020 and daily-additional counts of sick posts and other COVID-19 posts from November 10, 2019 to March 11, 2020 (*N*=122). 

**Figure 4:**
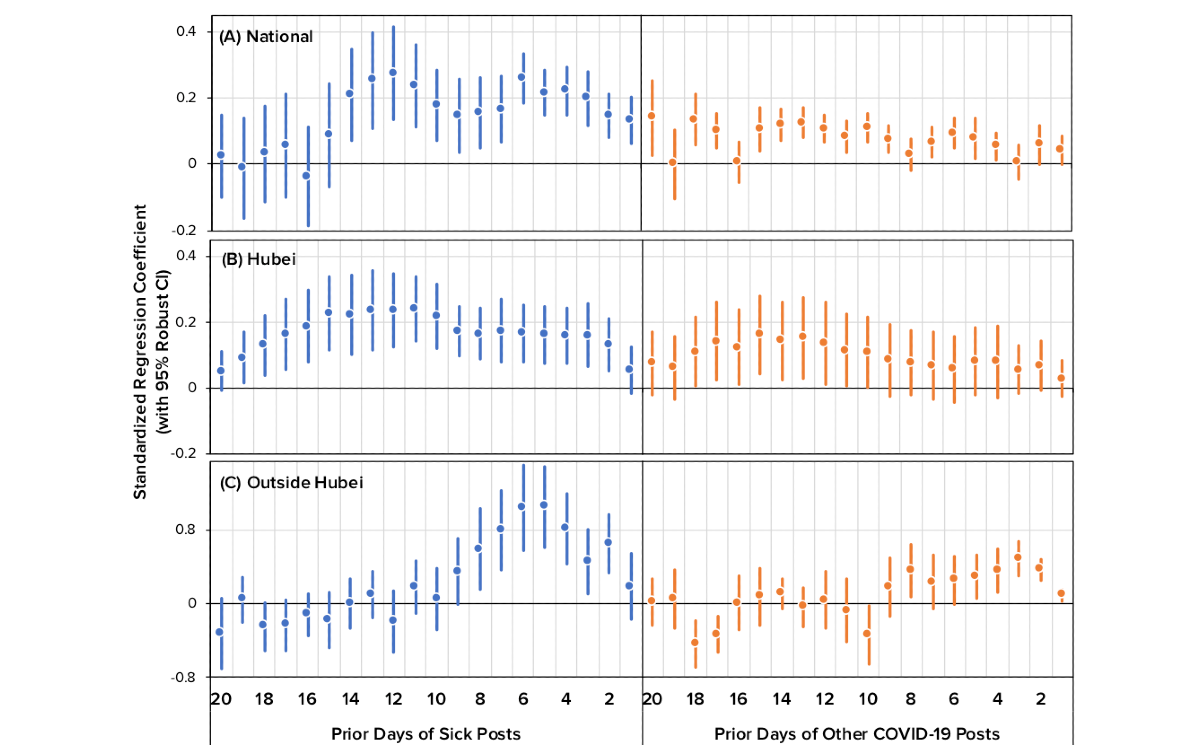
Standardized estimates of Granger causality for time-lagged, daily-additional Weibo posts (sick posts and other COVID-19 posts) predicting daily-additional cases.

Figure 4A summarizes the estimates of Granger causality for sick posts predicting new COVID-19 cases with standardized regression coefficients (see Table G in Multimedia Appendix 1 for all estimated parameters). Particularly, one standard deviation of increase in the daily-additional sick posts (1 sick post per 1 million posts) predicted a 0.133 (95% CI 0.065-0.201) to 0.275 (95% CI 0.134-0.416) standard deviation of the increase in the daily-additional new cases 1-14 days in advance. After including the 20 lags of sick posts, the adjusted *R^2^* value of the model increased by 0.128, suggesting that sick posts could explain an additional 12.8% of the variance of daily-additional new cases beyond the autoregressive terms and intervention effects.

Furthermore, we estimated the relationship between other COVID-19 post counts and daily-additional new cases using the same linear model. Figure 4A further illustrates the standardized estimates. Compared with sick posts, other COVID-19 posts were weaker signals of future case counts, as demonstrated by their smaller standard regression coefficients. This indicates that Weibo posts that discussed some aspect of COVID-19 but did not explicitly report a person’s symptoms or diagnosis had lower forecasting power than sick posts.  

To corroborate the above results, we tested the Granger causality of sick posts on cases within Hubei and outside Hubei (see Table H in Multimedia Appendix 1). Within Hubei, the results generally agreed with the national pattern mentioned above. Daily-additional sick posts predicted daily-additional new cases in Hubei up to 19 days in advance, as illustrated in Figure 4B. In contrast, other COVID-19 posts had fewer lag terms that could forecast new cases. Outside Hubei, the predictive pattern of sick posts was similar to the national pattern despite a limited time range: sick posts could forecast new cases 2 to 8 days in advance (see Figure 4C).

## Discussion

### Principal Findings

The novel coronavirus causing COVID-19 is a new pathogen in the human reservoir. It poses an extraordinary challenge for public health systems worldwide because screening and diagnostic tests must be developed from scratch. Even when such tests eventually become available, testing capacity is often severely limited; this can fuel the outbreak, as many patients can unknowingly infect other people. Based on approximately 15 million COVID-19-related Weibo posts between November 1, 2019 and March 31, 2020, we developed a supervised machine learning classifier to identify “sick posts,” in which a user reports their own or other people’s symptoms and diagnosis of COVID-19. Using the officially reported daily case counts as the outcome, our work shows that sick posts significantly predict daily cases up to 14 days ahead of official statistics. This finding confirms prior research that social media data can be usefully applied to nowcasting and forecasting emerging infectious diseases such as COVID-19 [22,34]. 

One of the greatest challenges of digital disease surveillance is identifying true disease signals, especially when facing the deluge of social media activity that resulted from COVID-19 mitigation measures [12,34-36]. Our finding that sick posts have greater predictive power than other COVID-19 posts shows that not all social media data are equally informative. Specifically, COVID-19 has dramatically disrupted everyday life; due to the pandemic, people are sheltering in place and increasingly communicating with others via social media. As shown in prior work [18] as well as in our data set, the majority of COVID-19–related chatter on Weibo reflected public awareness of COVID-19 rather than actual symptom reports. Most previous studies took rather coarse-grained approaches, relying primarily on either aggregated search query data or social media data retrieved from limited keyword searches [19,22]. In our work, we gathered the largest, most comprehensive, and most granular collection of social media data related to COVID-19 in the Chinese language. More importantly, we demonstrate a viable method to separate valid signals from noise using reports of symptoms and diagnosis, which makes a significant contribution to the literature on digital surveillance. 

Another important finding is that while the predictive power of sick posts on daily case counts holds true for both Hubei and non-Hubei regions, the effect sizes vary. Being the epicenter of the outbreak, Hubei province experienced extreme testing shortages during the early stage of the study period. As a result, many Hubei residents turned to social media sites such as Weibo to seek help for testing and medical care. In contrast, social media help-seeking activities were uncommon in other parts of China, where testing and health care resources were much more adequate. Taking these regional variations into account, we still observed predictive signals of sick posts on case counts, suggesting that the predictive power of sick posts was robust against testing delays. Further, the variations in the effect estimates show that the predictive power of social media data may vary across different geographic areas, with different levels of preparedness, and at different stages of the outbreak. Future studies based on longer periods of data monitoring could explore the temporal and spatial variations of COVID-19 social media surveillance efficacy in more depth.

Our work has broad public health implications. The high speed and low cost of social media surveillance can be especially useful in the early stages of the COVID-19 outbreak to inform containment and mitigation efforts when they are most cost-effective. For countries and regions where public health infrastructures do not allow for widespread screening and diagnostic tests, social media disease surveillance provides much-needed information for public health agencies to model the trajectories of the outbreak and to make swift decisions about allocation of resources such as hospital beds, ventilators, and personal protective equipment. 

Another advantage of social media surveillance is that it can be performed from a distance. As COVID-19 continues to spread worldwide, countries lacking testing and screening infrastructures will become “dark spots,” endangering their own citizens as well as the entire world. It is imperative that international organizations such as the World Health Organization integrate such data into their outbreak forecasting management practices to mobilize and coordinate relief efforts to help combat COVID-19. 

### Limitations

This study has several limitations. First, Weibo posts were retrieved retrospectively rather than in real time; therefore, deleted or censored posts were absent from our data set. However, we have no reason to believe that deletion or censorship favored “sick posts” in measurable ways. In fact, a recent study on Weibo censorship from December 2019-February 2020 shows that only 1.7/1000 Weibo posts were censored; also, these censored posts generally pertained to the missteps in the government’s COVID-19 response, not individual reports of symptoms and diagnoses [37]. Therefore, our results should not be affected by censorship. Second, as some studies suggest [38-40], confirmed COVID-19 case counts published by China CDC may underestimate the actual counts, due in part to limits in testing capacity and the existence of asymptomatic carriers. Still, the data here represent the best-known data of confirmed case counts, and our models rely on trends and changes in these case counts rather than the actual numbers. Third, it is important to acknowledge that sick posts as disease signals are not without noise because Weibo users who reported COVID-19 symptoms were not necessarily clinically diagnosed with COVID-19; Weibo users may not speak the truth; and Weibo users may “overreport” (posting about their symptoms or diagnoses multiple times) or “underreport” (not posting despite their symptoms or diagnoses) for a variety of reasons. Such inaccuracies are inherent in user-generated social media data and widely exist in all infoveillance studies. However, it should be noted that the goal of infoveillance has never been to achieve one-for-one matching between social media posts and clinical cases. Rather, infoveillance approaches strive to mine useful early signals from social media and internet data as a supplement to conventional surveillance efforts. Despite this noise, we still found that sick posts predicted COVID-19 case counts, indicating the validity of this signal in reflecting disease spread in the population.

### Conclusions

The threats of COVID-19 and other infectious diseases are likely to recur in the future. Reports of symptoms and diagnoses on social media during emerging disease outbreaks send invaluable warning signals to the public. Researchers and disease control agencies should pay close attention to the social media infosphere. In addition to monitoring overall search and posting activities, it is crucial to sift through the contents and efficiently separate true signals from noise. Our main findings highlight the importance of using rigorous procedures and understanding information sharing behaviors to obtain quality disease signals. Future studies based on longer periods of data monitoring could explore the time and spatial diffusions of COVID-19 in more depth. A more detailed examination of post contents reporting restraints in information or medical resources will be helpful in developing local outbreak responses. 

## Appendix

Multimedia Appendix 1 Supplementary tables.

## Abbreviations

API: application programming interface
China CDC: Chinese Center for Disease Control and Prevention
COVID-19: coronavirus disease
