# Supplementary material for: Using Reports of Symptoms and Diagnoses on Social Media to Predict COVID-19 Case Counts in Mainland China: Observational Infoveillance Study
Source: J Med Internet Res. 2020 May 28;22(5):e19421. doi: 10.2196/19421 (PMC7257484; doi:10.2196/19421)
Supplement: Multimedia Appendix 1 [file jmir_v22i5e19421_app1.docx]

**Using Reports of Own and Others’ Symptoms and Diagnosis on Social Media to Predict COVID-19 Case Counts: Observational Infoveillance Study in Mainland China**

**Multimedia Appendix**

[Table A. COVID-19 related keywords used to retrieve Weibo posts](#_Toc40691036)

[Table B. Summaries of modified Dickey-Fuller *t* tests for a unit root (without trend) in the time series of new cases, sick posts, or other COVID-19 posts in mainland China (*N* = 122)](#_Toc40691037)

[Table C. Model comparisons for sick post or other COVID-19 post (difference scores) predicting new cases (difference scores) in mainland China with varying lag terms (*N* = 122)](#_Toc40691038)

[Table D. Model summaries for sick posts (difference scores) predicting new cases (difference scores) in mainland China with varying lag terms (*N* = 122)](#_Toc40691039)

[Table E. Model summaries for other COVID-19 posts (difference scores) predicting new cases (difference scores) in mainland China with varying lag terms (*N* = 122)](#_Toc40691040)

[Table F. Model comparisons for sick posts or other COVID-19 posts (difference scores) predicting new cases (difference scores) in mainland China with varying linear decay rates of the effect of the changed diagnostic criteria on February 12^th^, 2020 (*N* = 122)](#_Toc40691041)

[Table G. Model summaries of sick posts or other COVID-19 posts (difference scores) predicting new cases (difference scores) in mainland China, including a baseline model without effects of social media posts (*N* = 122)](#_Toc40691042)

[Table H. Model summaries of sick posts or other COVID-19 posts (difference scores) predicting new cases (difference scores) within or outside Hubei (*N* = 122)](#_Toc40691043)

Table A. COVID-19 related keywords used to retrieve Weibo posts

| **Keyword** | **Translation** |  | **Keyword** | **Translation** |
| --- | --- | --- | --- | --- |
| 武汉肺炎 | Wuhan pneumonia |  | 潜伏期 | Incubation period |
| 新型冠状病毒肺炎 | COVID-19 |  | 北京 AND 病例 | Beijing AND Cases |
| 不明原因肺炎 | Pneumonia of unknown cause |  | 天津 AND 病例 | Tianjin AND Cases |
| 肺炎疫情 | Pneumonia outbreak |  | 河北 AND 病例 | Hebei AND Cases |
| 野味肺炎 | Wildlife pneumonia |  | 辽宁 AND 病例 | Liaoning AND Cases |
| 新型冠状病毒 AND 确诊 | Novel coronavirus AND Confirmed infected |  | 上海 AND 病例 | Shanghai AND Cases |
| 感染人数 | Number of infected cases |  | 江苏 AND 病例 | Jiangsu AND Cases |
| 出门 AND 戴口罩 | Going out AND Wear mask |  | 浙江 AND 病例 | Zhejiang AND Cases |
| N95 AND 口罩 | N95 AND Mask |  | 福建 AND 病例 | Fujian AND Cases |
| 3M AND 口罩 | 3M AND Mask |  | 山东 AND 病例 | Shandong AND Cases |
| KN95 AND 口罩 | KN95 AND Mask |  | 广东 AND 病例 | Guangdong AND Cases |
| 大众畜牧野味店 | Dazhong wildlife restaurant |  | 海南 AND 病例 | Hainan AND Cases |
| 口罩 | Mask |  | 山西 AND 病例 | Shanxi AND Cases |
| 新肺炎 | Novel pneumonia |  | 内蒙古 AND 病例 | Inner Mongolia AND Cases |
| 华南野生市场 | South China wild market |  | 吉林 AND 病例 | Jilin AND Cases |
| 冠状肺炎 | Corona pneumonia |  | 黑龙江 AND 病例 | Heilongjiang AND Cases |
| 武汉病毒所 | Wuhan Institute of Virology |  | 安徽 AND 病例 | Anhui AND Cases |
| China AND CDC | China AND Center for Disease Control and Prevention |  | 江西 AND 病例 | Jiangxi AND Cases |
| 中国疾病预防控制中心 | Chinese Center for Disease Control and Prevention |  | 河南 AND 病例 | Henan AND Cases |
| #2019nCoV | ·· |  | 湖北 AND 病例 | Hubei AND Cases |
| 双黄连 AND 抢购 | Shuanghuanglian AND Rush to buy |  | 湖南 AND 病例 | Hunan AND Cases |
| 双黄连 AND 售磬 | Shuanghuanglian AND Sold out |  | 广西 AND 病例 | Guangxi AND Cases |
| 武汉卫健委 | Wuhan Municipal Health Committee |  | 四川 AND 病例 | Sichuan AND Cases |
| 湖北卫健委 | Health Commission of Hubei Province |  | 贵州 AND 病例 | Guizhou AND Cases |
| 肺炎 | Pneumonia |  | 云南 AND 病例 | Yunnan AND Cases |
| 疫情 | Epidemic outbreak |  | 西藏 AND 病例 | Tibet AND Cases |
| 隔离 | Quarantine |  | 陕西 AND 病例 | Shanxi AND Cases |
| 火神山 | Huoshen Shan hospital |  | 甘肃 AND 病例 | Gansu AND Cases |
| 雷神山 | Leishen Shan hospital |  | 青海 AND 病例 | Qinghai AND Cases |
| 钟南山 | Zhong Nanshan |  | 宁夏 AND 病例 | Ningxia AND Cases |
| 疫情防控 | Epidemic prevention and control |  | 新疆 AND 病例 | Xinjiang AND Cases |
| Coronavirus | ·· |  | 香港 AND 病例 | Hong Kong AND Cases |
| Remdesivir | ·· |  | 澳门 AND 病例 | Macau AND Cases |
| 瑞德西韦 | Remdesivir |  | 台湾 AND 病例 | Taiwan AND Cases |
| 新型肺炎 AND 死亡 | Novel coronavirus pneumonia AND Death |  | ECMO | Extracorporeal Membrane Oxygenation |
| 新型肺炎 AND 感染 | Novel coronavirus pneumonia AND Infection |  | 人工膜肺 | Extracorporeal membrane oxygenation |
| 新型冠状病毒 AND 感染 | Novel coronavirus AND Infection |  | 双盲测试 | Double blind test |
| 感染 AND 案例 | Infected AND Cases |  | 核酸检测 | Nucleic acid testing |
| 武汉 AND 封城 | Wuhan AND Lockdown |  | 疫苗 | Vaccine |
| 高福 | George Fu Gao |  | 小区出入证 | Community pass card |
| 王延轶 | Wang Yanyi |  | 战疫 | Anti-COVID-19 |
| 舒红兵 | Shu Hongbing |  | 抗疫 | Anti-COVID-19 |
| 协和医院 | Xiehe Hospital |  | 全国疫情 | Epidemic in China |
| 武汉 AND 隔离 | Wuhan AND Quarantine |  | 囤积口罩 | Hoarding mask |
| 医生 AND 李文亮 | Doctor AND Li Wenliang |  | 湖北卫健委 AND 免职 | Health commission of Hubei Province AND Remove from the position |
| 云监工 | Supervising work on cloud |  | 发热患者 | Fever patients |
| 武汉 AND 肺炎 AND 谣言 | Wuhan AND Pneumonia AND Rumors |  | 延迟开学 | Postpone the reopening of school |
| 8名 AND 散布武汉肺炎谣言 | Eight people AND Spread rumors of Wuhan pneumonia |  | 开学时间 AND 不得早于 | The start time of school AND Not earlier than |
| 武汉仁爱医院 | Wuhan Ren'ai Hospital |  | 累计死亡数 | Cumulative deaths |
| 黄冈 AND 新肺炎 | Huanggang AND Novel pneumonia |  | 疑似病例 | Suspicious cases |
| 黄冈 AND 新型冠状病毒 | Huanggang AND Novel coronavirus |  | 入户排查 | Household troubleshoot |
| 黄冈 AND 感染者 | Huanggang AND Infected cases |  | 武汉市慈善总会 | Wuhan Charity Federation |
| 孝感 AND 新肺炎 | Xiaogan AND Novel pneumonia |  | 防疫物资 | Epidemic control and prevention materials |
| 孝感 AND 新型冠状病毒 | Xiaogan AND Novel coronavirus |  | 捐赠物资 | Donation materials |
| 孝感 AND 感染者 | Xiaogan AND Infected cases |  | 俄罗斯 AND 捐赠 | Russia AND Donations |
| 居家隔离 | Isolated at home |  | 巴基斯坦 AND 捐赠 | Pakistan AND Donations |
| 隔离 AND 14天 | Isolation AND 14 days |  | 美国 AND 捐赠 | United States AND Donations |
| 潜伏期 AND 24天 | Incubation period AND 24 days |  | 日本 AND 捐赠 | Japan AND Donations |
| 潜伏期 AND 14天 | Incubation period AND 14 days |  | MERS | Middle East Respiratory Syndrome |
| 新型肺炎 | Novel pneumonia |  | 中央赴湖北指导小组 | Delegation from central government to guide Hubei |
| 新型冠状病毒 | Novel coronavirus |  | 抗击 AND 新型肺炎 | Fight against AND COVID-19 |
| 国际公共卫生紧急事件 | International Public Health Emergencies |  | 支援武汉 | Give a hand to Wuhan |
| PHEIC | International Public Health Emergencies |  | 医用口罩 | Surgical mask |
| #nCoV | ·· |  | 武汉 AND 新增 | Wuhan AND Novel cases |
| 方舱医院 | FangCang Hospital |  | 临床诊断病例 | Clinically diagnosed cases |
| 一省包一市 | One province gives a hand to one Hubei city |  | 应勇 AND 湖北 | Ying Yong AND Hubei |
| 新冠肺炎 | Novel coronavirus pneumonia |  | 应勇 AND 上海 | Ying Yong AND Shanghai |
| 晋江毒王 | Super spreader of COVID-19 in Jinjiang |  | 蒋超良 AND 湖北 | Jiang Chaoliang AND Hubei |
| 超级传播者 | Super spreader |  | SARS-CoV-2 | ·· |
| 湖北 AND王晓东 | Hubei AND Wang Xiaodong |  | 武汉 AND 死亡病例 | Wuhan AND Death cases |
| 蒋超良 | Jiang Chaoliang |  | 武汉 AND 感染病例 | Wuhan AND Infection cases |
| #武汉肺炎 | #Wuhan pneumonia |  | 湖北 AND 死亡病例 | Hubei AND Death cases |
| 武汉 AND 李文亮 | Wuhan AND Li Wenliang |  | 湖北 AND 感染病例 | Hubei AND Infected cases |
| 武汉 AND 李医生 | Wuhan AND Dr. Li |  | 中国 AND 死亡病例 | China AND Death cases |
| 武汉 AND 疫情 | Wuhan AND Epidemic |  | 中国 AND 感染病例 | China AND Infected cases |
| 国家疾控中心 | Chinese Center for Disease Control and Prevention |  | 企业复工 | Enterprise work resuming |
| 武汉 AND 疫苗 | Wuhan AND Vaccine |  | 中小企业 AND 困境 | Small and medium-sized enterprise AND Dilemma |
| 管轶 | Guan Yi |  | 超市采购 | Supermarket Purchase |
| 张晋 AND 卫健委 | Zhang Jin AND Health Commission |  | 西贝 | Xibei |
| 张晋 AND 卫生健康委员会 | Zhang Jin AND Health Commission |  | 武汉 AND 征用宿舍 | Wuhan AND Requisitioned students’ dormitory |
| 刘英姿 AND 卫健委 | Liu Yingzi AND Health Commission |  | 周佩仪 | Zhou Peiyi |
| 刘英姿 AND 卫生健康委员会 | Liu Yingzi AND Health Commission |  | 武汉中心医院 | The Central Hospital of Wuhan |
| 王贺胜 AND 卫健委 | Wang Hesheng AND Health Commission |  | 武汉病毒研究 | Virology research in Wuhan |
| 王贺胜 AND 卫生健康委员会 | Wang Hesheng AND Health Commission |  |  |  |

##

## Table B. Summaries of modified Dickey-Fuller *t* tests for a unit root (without trend) in the time series of new cases, sick posts, or other COVID-19 posts in mainland China (*N* = 122)

|  | **New Cases** | | | | | | | | |  | **Sick Posts** | | | | | | | | |  | **Other COVID-19 Posts** | | | | | | | | |
| --- | --- | --- | --- | --- | --- | --- | --- | --- | --- | --- | --- | --- | --- | --- | --- | --- | --- | --- | --- | --- | --- | --- | --- | --- | --- | --- | --- | --- | --- |
|  | **Raw Counts** | | | |  | **Difference Scores  (Daily-Additional Counts)** | | | |  | **Raw Counts** | | | |  | **Difference Scores  (Daily-Additional Counts)** | | | |  | **Raw Counts** | | | |  | **Difference Scores  (Daily-Additional Counts)** | | | |
| **Max  Lags** | ***t*** | **1%**  **CV** | **5%**  **CV** | **10%**  **CV** |  | ***t*** | **1%**  **CV** | **5%**  **CV** | **10%**  **CV** |  | ***t*** | **1%**  **CV** | **5%**  **CV** | **10%**  **CV** |  | ***t*** | **1%**  **CV** | **5%**  **CV** | **10%**  **CV** |  | ***t*** | **1%**  **CV** | **5%**  **CV** | **10%**  **CV** |  | ***t*** | **1%**  **CV** | **5%**  **CV** | **10%**  **CV** |
| 29 | -1.212 | -2.597 | -1.899 | -1.557 |  | -2.087 | -2.597 | -1.899 | -1.557 |  | -1.024 | -2.597 | -1.899 | -1.557 |  | -1.993 | -2.597 | -1.899 | -1.557 |  | -1.075 | -2.597 | -1.899 | -1.557 |  | -1.833 | -2.597 | -1.899 | -1.557 |
| 28 | -1.234 | -2.597 | -1.887 | -1.551 |  | -2.139 | -2.597 | -1.887 | -1.551 |  | -0.989 | -2.597 | -1.887 | -1.551 |  | -1.940 | -2.597 | -1.887 | -1.551 |  | -1.207 | -2.597 | -1.887 | -1.551 |  | -1.829 | -2.597 | -1.887 | -1.551 |
| 27 | -1.264 | -2.597 | -1.879 | -1.548 |  | -2.186 | -2.597 | -1.879 | -1.548 |  | -0.962 | -2.597 | -1.879 | -1.548 |  | -2.091 | -2.597 | -1.879 | -1.548 |  | -1.134 | -2.597 | -1.879 | -1.548 |  | -1.677 | -2.597 | -1.879 | -1.548 |
| 26 | -1.304 | -2.597 | -1.873 | -1.546 |  | -2.222 | -2.597 | -1.873 | -1.546 |  | -1.024 | -2.597 | -1.873 | -1.546 |  | -2.250 | -2.597 | -1.873 | -1.546 |  | -1.110 | -2.597 | -1.873 | -1.546 |  | -1.839 | -2.597 | -1.873 | -1.546 |
| 25 | -1.349 | -2.597 | -1.869 | -1.547 |  | -2.242 | -2.597 | -1.869 | -1.547 |  | -1.070 | -2.597 | -1.869 | -1.547 |  | -2.209 | -2.597 | -1.869 | -1.547 |  | -1.206 | -2.597 | -1.869 | -1.547 |  | -1.942 | -2.597 | -1.869 | -1.547 |
| 24 | -1.402 | -2.597 | -1.868 | -1.549 |  | -2.252 | -2.597 | -1.868 | -1.549 |  | -1.110 | -2.597 | -1.868 | -1.549 |  | -2.206 | -2.597 | -1.868 | -1.549 |  | -1.124 | -2.597 | -1.868 | -1.549 |  | -1.842 | -2.597 | -1.868 | -1.549 |
| 23 | -1.464 | -2.597 | -1.868 | -1.553 |  | -2.249 | -2.597 | -1.868 | -1.553 |  | -1.091 | -2.597 | -1.868 | -1.553 |  | -2.216 | -2.597 | -1.868 | -1.553 |  | -1.195 | -2.597 | -1.868 | -1.553 |  | -2.047 | -2.597 | -1.868 | -1.553 |
| 22 | -1.521 | -2.597 | -1.871 | -1.558 |  | -2.230 | -2.597 | -1.871 | -1.558 |  | -1.251 | -2.597 | -1.871 | -1.558 |  | -2.357 | -2.597 | -1.871 | -1.558 |  | -1.267 | -2.597 | -1.871 | -1.558 |  | -1.990 | -2.597 | -1.871 | -1.558 |
| 21 | -1.578 | -2.597 | -1.875 | -1.565 |  | -2.219 | -2.597 | -1.875 | -1.565 |  | -1.371 | -2.597 | -1.875 | -1.565 |  | -2.135 | -2.597 | -1.875 | -1.565 |  | -1.411 | -2.597 | -1.875 | -1.565 |  | -1.934 | -2.597 | -1.875 | -1.565 |
| 20 | -1.641 | -2.597 | -1.881 | -1.574 |  | -2.206 | -2.597 | -1.881 | -1.574 |  | -1.290 | -2.597 | -1.881 | -1.574 |  | -2.009 | -2.597 | -1.881 | -1.574 |  | -1.444 | -2.597 | -1.881 | -1.574 |  | -1.780 | -2.597 | -1.881 | -1.574 |
| 19 | -1.673 | -2.597 | -1.888 | -1.583 |  | -2.184 | -2.597 | -1.888 | -1.583 |  | -1.428 | -2.597 | -1.888 | -1.583 |  | -2.206 | -2.597 | -1.888 | -1.583 |  | -1.238 | -2.597 | -1.888 | -1.583 |  | -1.777 | -2.597 | -1.888 | -1.583 |
| 18 | -1.697 | -2.597 | -1.897 | -1.594 |  | -2.200 | -2.597 | -1.897 | -1.594 |  | -1.578 | -2.597 | -1.897 | -1.594 |  | -2.056 | -2.597 | -1.897 | -1.594 |  | -1.355 | -2.597 | -1.897 | -1.594 |  | -2.139 | -2.597 | -1.897 | -1.594 |
| 17 | -1.693 | -2.597 | -1.907 | -1.605 |  | -2.229 | -2.597 | -1.907 | -1.605 |  | -1.712 | -2.597 | -1.907 | -1.605 |  | -1.905 | -2.597 | -1.907 | -1.605 |  | -1.388 | -2.597 | -1.907 | -1.605 |  | -2.012 | -2.597 | -1.907 | -1.605 |
| 16 | -1.663 | -2.597 | -1.918 | -1.617 |  | -2.298 | -2.597 | -1.918 | -1.617 |  | -1.629 | -2.597 | -1.918 | -1.617 |  | -1.787 | -2.597 | -1.918 | -1.617 |  | -1.548 | -2.597 | -1.918 | -1.617 |  | -2.019 | -2.597 | -1.918 | -1.617 |
| 15 | -1.630 | -2.597 | -1.931 | -1.630 |  | -2.414 | -2.597 | -1.931 | -1.630 |  | -1.600 | -2.597 | -1.931 | -1.630 |  | -1.911 | -2.597 | -1.931 | -1.630 |  | -1.287 | -2.597 | -1.931 | -1.630 |  | -1.851 | -2.597 | -1.931 | -1.630 |
| 14 | -1.691 | -2.597 | -1.943 | -1.644 |  | -2.552 | -2.597 | -1.943 | -1.644 |  | -1.466 | -2.597 | -1.943 | -1.644 |  | -1.986 | -2.597 | -1.943 | -1.644 |  | -1.150 | -2.597 | -1.943 | -1.644 |  | -2.299 | -2.597 | -1.943 | -1.644 |
| 13 | -1.660 | -2.597 | -1.957 | -1.658 |  | -2.551 | -2.597 | -1.957 | -1.658 |  | -1.284 | -2.597 | -1.957 | -1.658 |  | -2.224 | -2.597 | -1.957 | -1.658 |  | -1.096 | -2.597 | -1.957 | -1.658 |  | -2.701 | -2.597 | -1.957 | -1.658 |
| 12 | -1.628 | -2.597 | -1.971 | -1.672 |  | -2.701 | -2.597 | -1.971 | -1.672 |  | -1.304 | -2.597 | -1.971 | -1.672 |  | -2.643 | -2.597 | -1.971 | -1.672 |  | -1.497 | -2.597 | -1.971 | -1.672 |  | -3.004 | -2.597 | -1.971 | -1.672 |
| 11 | -1.583 | -2.597 | -1.986 | -1.686 |  | -2.880 | -2.597 | -1.986 | -1.686 |  | -1.180 | -2.597 | -1.986 | -1.686 |  | -2.725 | -2.597 | -1.986 | -1.686 |  | -1.495 | -2.597 | -1.986 | -1.686 |  | -2.276 | -2.597 | -1.986 | -1.686 |
| 10 | -1.526 | -2.597 | -2.000 | -1.701 |  | -3.122 | -2.597 | -2.000 | -1.701 |  | -1.206 | -2.597 | -2.000 | -1.701 |  | -3.199 | -2.597 | -2.000 | -1.701 |  | -1.397 | -2.597 | -2.000 | -1.701 |  | -2.353 | -2.597 | -2.000 | -1.701 |
| 9 | -1.475 | -2.597 | -2.015 | -1.715 |  | -3.456 | -2.597 | -2.015 | -1.715 |  | -1.449 | -2.597 | -2.015 | -1.715 |  | -3.349 | -2.597 | -2.015 | -1.715 |  | -1.276 | -2.597 | -2.015 | -1.715 |  | -2.619 | -2.597 | -2.015 | -1.715 |
| 8 | -1.455 | -2.597 | -2.030 | -1.729 |  | -3.881 | -2.597 | -2.030 | -1.729 |  | -1.686 | -2.597 | -2.030 | -1.729 |  | -2.937 | -2.597 | -2.030 | -1.729 |  | -1.126 | -2.597 | -2.030 | -1.729 |  | -3.022 | -2.597 | -2.030 | -1.729 |
| 7 | -1.416 | -2.597 | -2.044 | -1.743 |  | -4.351 | -2.597 | -2.044 | -1.743 |  | -1.477 | -2.597 | -2.044 | -1.743 |  | -2.623 | -2.597 | -2.044 | -1.743 |  | -1.244 | -2.597 | -2.044 | -1.743 |  | -3.707 | -2.597 | -2.044 | -1.743 |
| 6 | -1.515 | -2.597 | -2.059 | -1.757 |  | -5.104 | -2.597 | -2.059 | -1.757 |  | -1.423 | -2.597 | -2.059 | -1.757 |  | -3.149 | -2.597 | -2.059 | -1.757 |  | -1.211 | -2.597 | -2.059 | -1.757 |  | -3.618 | -2.597 | -2.059 | -1.757 |
| 5 | -1.592 | -2.597 | -2.073 | -1.770 |  | -5.563 | -2.597 | -2.073 | -1.770 |  | -1.220 | -2.597 | -2.073 | -1.770 |  | -3.489 | -2.597 | -2.073 | -1.770 |  | -1.338 | -2.597 | -2.073 | -1.770 |  | -4.071 | -2.597 | -2.073 | -1.770 |
| 4 | -1.729 | -2.597 | -2.086 | -1.782 |  | -6.413 | -2.597 | -2.086 | -1.782 |  | -1.088 | -2.597 | -2.086 | -1.782 |  | -4.526 | -2.597 | -2.086 | -1.782 |  | -1.345 | -2.597 | -2.086 | -1.782 |  | -4.025 | -2.597 | -2.086 | -1.782 |
| 3 | -2.049 | -2.597 | -2.098 | -1.793 |  | -7.564 | -2.597 | -2.098 | -1.793 |  | -1.214 | -2.597 | -2.098 | -1.793 |  | -6.065 | -2.597 | -2.098 | -1.793 |  | -1.574 | -2.597 | -2.098 | -1.793 |  | -4.433 | -2.597 | -2.098 | -1.793 |
| 2 | -2.443 | -2.597 | -2.110 | -1.804 |  | -8.575 | -2.597 | -2.110 | -1.804 |  | -1.146 | -2.597 | -2.110 | -1.804 |  | -6.686 | -2.597 | -2.110 | -1.804 |  | -1.179 | -2.597 | -2.110 | -1.804 |  | -4.142 | -2.597 | -2.110 | -1.804 |
| 1 | -3.221 | -2.597 | -2.120 | -1.814 |  | -10.731 | -2.597 | -2.120 | -1.814 |  | -1.642 | -2.597 | -2.120 | -1.814 |  | -10.739 | -2.597 | -2.120 | -1.814 |  | -1.187 | -2.597 | -2.120 | -1.814 |  | -6.801 | -2.597 | -2.120 | -1.814 |

Note. CV = critical value.

## Table C. Model comparisons for sick post or other COVID-19 post (difference scores) predicting new cases (difference scores) in mainland China with varying lag terms (*N* = 122)

|  | **Cases Regressed on Sick Posts** | | | |  | **Cases Regressed on Other COVID-19 Posts** | | | |
| --- | --- | --- | --- | --- | --- | --- | --- | --- | --- |
| **Max Lags** | **Adjusted R^2^ (Δ)** | **AIC** | **BIC** | **Model df** |  | **Adjusted R^2^ (Δ)** | **AIC** | **BIC** | **Model df** |
| 1 | 0.766 (–) | 1963.334 | 1974.550 | 3 |  | 0.746 (.) | 1973.108 | 1984.324 | 3 |
| 2 | 0.820 (0.055) | 1932.878 | 1949.702 | 5 |  | 0.804 (0.058) | 1943.581 | 1960.405 | 5 |
| 3 | 0.870 (0.050) | 1895.064 | 1917.497 | 7 |  | 0.831 (0.027) | 1927.413 | 1949.845 | 7 |
| 4 | 0.883 (0.013) | 1884.280 | 1912.320 | 9 |  | 0.843 (0.012) | 1920.511 | 1948.551 | 9 |
| 5 | 0.900 (0.017) | 1866.985 | 1900.633 | 11 |  | 0.846 (0.004) | 1919.511 | 1953.160 | 11 |
| 6 | 0.929 (0.029) | 1826.665 | 1865.921 | 13 |  | 0.850 (0.004) | 1918.410 | 1957.666 | 13 |
| 7 | 0.934 (0.005) | 1819.595 | 1864.460 | 15 |  | 0.853 (0.003) | 1917.858 | 1962.723 | 15 |
| 8 | 0.936 (0.002) | 1817.837 | 1868.309 | 17 |  | 0.852 (-0.001) | 1920.235 | 1970.708 | 17 |
| 9 | 0.936 (0.000) | 1820.310 | 1876.390 | 19 |  | 0.853 (0.001) | 1921.165 | 1977.245 | 19 |
| 10 | 0.936 (0.001) | 1820.552 | 1882.241 | 21 |  | 0.862 (0.010) | 1914.515 | 1976.203 | 21 |
| 11 | 0.939 (0.002) | 1817.247 | 1884.544 | 23 |  | 0.868 (0.006) | 1910.896 | 1978.192 | 23 |
| 12 | 0.946 (0.008) | 1802.692 | 1875.596 | 25 |  | 0.882 (0.014) | 1898.750 | 1971.655 | 25 |
| 13 | 0.949 (0.003) | 1797.454 | 1875.966 | 27 |  | 0.887 (0.005) | 1894.797 | 1973.310 | 27 |
| 14 | 0.957 (0.008) | 1777.530 | 1861.651 | 29 |  | 0.895 (0.008) | 1887.346 | 1971.466 | 29 |
| 15 | 0.964 (0.007) | 1757.691 | 1847.420 | 31 |  | 0.913 (0.019) | 1864.928 | 1954.657 | 31 |
| 16 | 0.964 (0.000) | 1758.488 | 1853.824 | 33 |  | 0.917 (0.004) | 1861.019 | 1956.355 | 33 |
| 17 | 0.966 (0.002) | 1751.786 | 1852.731 | 35 |  | 0.930 (0.013) | 1842.193 | 1943.138 | 35 |
| 18 | 0.969 (0.002) | 1743.840 | 1850.392 | 37 |  | 0.942 (0.012) | 1819.755 | 1926.308 | 37 |
| 19 | 0.970 (0.001) | 1742.406 | 1854.566 | 39 |  | 0.947 (0.005) | 1809.707 | 1921.868 | 39 |
| 20 | 0.970 (0.000) | 1741.721 | 1859.490 | 41 |  | 0.954 (0.007) | 1792.342 | 1910.110 | 41 |
| 21 | 0.973 (0.003) | 1731.055 | 1854.432 | 43 |  | 0.960 (0.006) | 1776.301 | 1899.678 | 43 |
| 22 | 0.973 (0.000) | 1729.606 | 1858.591 | 45 |  | 0.964 (0.004) | 1765.199 | 1894.184 | 45 |
| 23 | 0.973 (0.000) | 1731.977 | 1866.570 | 47 |  | 0.972 (0.008) | 1737.516 | 1872.109 | 47 |
| 24 | 0.974 (0.001) | 1726.073 | 1866.274 | 49 |  | 0.980 (0.009) | 1694.121 | 1834.322 | 49 |
| 25 | 0.978 (0.004) | 1706.914 | 1852.723 | 51 |  | 0.984 (0.004) | 1665.321 | 1811.130 | 51 |
| 26 | 0.982 (0.004) | 1683.031 | 1834.448 | 53 |  | 0.986 (0.001) | 1655.912 | 1807.329 | 53 |
| 27 | 0.983 (0.001) | 1676.765 | 1833.790 | 55 |  | 0.988 (0.002) | 1637.829 | 1794.854 | 55 |
| 28 | 0.984 (0.001) | 1670.747 | 1833.380 | 57 |  | 0.989 (0.001) | 1627.644 | 1790.277 | 57 |
| 29 | 0.986 (0.002) | 1650.172 | 1818.414 | 59 |  | 0.988 (0.000) | 1630.254 | 1798.496 | 59 |

## Table D. Model summaries for sick posts (difference scores) predicting new cases (difference scores) in mainland China with varying lag terms (*N* = 122)

|  | **Model 1 (max lag = 5)** | | |  | **Model 2 (max lag = 10)** | | |  | **Model 3 (max lag = 15)** | | |  | **Model 4 (max lag = 20)** | | |  | **Model 5 (max lag = 25)** | | |  | **Model 6 (max lag = 29)** | | |
| --- | --- | --- | --- | --- | --- | --- | --- | --- | --- | --- | --- | --- | --- | --- | --- | --- | --- | --- | --- | --- | --- | --- | --- |
|  | ***B*** | ***SE*** | ***p*** |  | ***B*** | ***SE*** | ***p*** |  | ***B*** | ***SE*** | ***p*** |  | ***B*** | ***SE*** | ***p*** |  | ***B*** | ***SE*** | ***p*** |  | ***B*** | ***SE*** | ***p*** |
| Intercept | -114.238 | 44.247 | 0.011 |  | -99.007 | 35.435 | 0.006 |  | -102.012 | 26.627 | 0.000 |  | -99.941 | 24.650 | 0.000 |  | -91.168 | 21.238 | 0.000 |  | -93.310 | 16.901 | 0.000 |
| Change of Diagnosis Criteria | 13703.510 | 565.800 | 0.000 |  | 11712.520 | 553.424 | 0.000 |  | 11649.240 | 447.200 | 0.000 |  | 11476.130 | 612.788 | 0.000 |  | 10474.620 | 630.933 | 0.000 |  | 10594.030 | 550.274 | 0.000 |
| **Daily Additional New Cases** |  |  |  |  |  |  |  |  |  |  |  |  |  |  |  |  |  |  |  |  |  |  |  |
| Lag = 1 | -0.620 | 0.036 | 0.000 |  | -0.682 | 0.045 | 0.000 |  | -0.746 | 0.037 | 0.000 |  | -0.686 | 0.049 | 0.000 |  | -0.695 | 0.051 | 0.000 |  | -0.739 | 0.045 | 0.000 |
| Lag = 2 | -0.490 | 0.043 | 0.000 |  | -0.552 | 0.059 | 0.000 |  | -0.669 | 0.050 | 0.000 |  | -0.569 | 0.062 | 0.000 |  | -0.521 | 0.066 | 0.000 |  | -0.644 | 0.060 | 0.000 |
| Lag = 3 | -0.356 | 0.045 | 0.000 |  | -0.478 | 0.062 | 0.000 |  | -0.657 | 0.055 | 0.000 |  | -0.582 | 0.064 | 0.000 |  | -0.619 | 0.068 | 0.000 |  | -0.727 | 0.062 | 0.000 |
| Lag = 4 | -0.178 | 0.041 | 0.000 |  | -0.360 | 0.060 | 0.000 |  | -0.605 | 0.058 | 0.000 |  | -0.524 | 0.063 | 0.000 |  | -0.623 | 0.074 | 0.000 |  | -0.745 | 0.065 | 0.000 |
| Lag = 5 | -0.100 | 0.036 | 0.006 |  | -0.256 | 0.050 | 0.000 |  | -0.613 | 0.061 | 0.000 |  | -0.544 | 0.062 | 0.000 |  | -0.589 | 0.075 | 0.000 |  | -0.753 | 0.065 | 0.000 |
| Lag = 6 |  |  |  |  | -0.140 | 0.053 | 0.009 |  | -0.578 | 0.069 | 0.000 |  | -0.511 | 0.072 | 0.000 |  | -0.579 | 0.078 | 0.000 |  | -0.745 | 0.070 | 0.000 |
| Lag = 7 |  |  |  |  | -0.110 | 0.052 | 0.037 |  | -0.573 | 0.072 | 0.000 |  | -0.465 | 0.081 | 0.000 |  | -0.480 | 0.087 | 0.000 |  | -0.715 | 0.081 | 0.000 |
| Lag = 8 |  |  |  |  | -0.058 | 0.044 | 0.192 |  | -0.543 | 0.069 | 0.000 |  | -0.429 | 0.082 | 0.000 |  | -0.390 | 0.088 | 0.000 |  | -0.693 | 0.087 | 0.000 |
| Lag = 9 |  |  |  |  | -0.048 | 0.036 | 0.183 |  | -0.474 | 0.060 | 0.000 |  | -0.377 | 0.081 | 0.000 |  | -0.383 | 0.081 | 0.000 |  | -0.649 | 0.083 | 0.000 |
| Lag = 10 |  |  |  |  | -0.034 | 0.030 | 0.256 |  | -0.339 | 0.045 | 0.000 |  | -0.284 | 0.081 | 0.001 |  | -0.271 | 0.082 | 0.002 |  | -0.445 | 0.081 | 0.000 |
| Lag = 11 |  |  |  |  |  |  |  |  | -0.297 | 0.044 | 0.000 |  | -0.317 | 0.079 | 0.000 |  | -0.345 | 0.080 | 0.000 |  | -0.478 | 0.075 | 0.000 |
| Lag = 12 |  |  |  |  |  |  |  |  | -0.262 | 0.043 | 0.000 |  | -0.275 | 0.082 | 0.001 |  | -0.271 | 0.084 | 0.002 |  | -0.402 | 0.075 | 0.000 |
| Lag = 13 |  |  |  |  |  |  |  |  | -0.185 | 0.036 | 0.000 |  | -0.169 | 0.079 | 0.035 |  | -0.034 | 0.085 | 0.689 |  | -0.198 | 0.075 | 0.010 |
| Lag = 14 |  |  |  |  |  |  |  |  | -0.088 | 0.028 | 0.002 |  | -0.137 | 0.067 | 0.043 |  | -0.048 | 0.079 | 0.540 |  | -0.187 | 0.074 | 0.014 |
| Lag = 15 |  |  |  |  |  |  |  |  | -0.051 | 0.024 | 0.035 |  | -0.143 | 0.047 | 0.003 |  | -0.174 | 0.081 | 0.034 |  | -0.223 | 0.076 | 0.004 |
| Lag = 16 |  |  |  |  |  |  |  |  |  |  |  |  | -0.121 | 0.045 | 0.009 |  | -0.095 | 0.083 | 0.256 |  | -0.156 | 0.075 | 0.042 |
| Lag = 17 |  |  |  |  |  |  |  |  |  |  |  |  | -0.125 | 0.046 | 0.008 |  | -0.111 | 0.083 | 0.183 |  | -0.130 | 0.075 | 0.087 |
| Lag = 18 |  |  |  |  |  |  |  |  |  |  |  |  | -0.113 | 0.040 | 0.006 |  | -0.086 | 0.080 | 0.286 |  | -0.113 | 0.071 | 0.119 |
| Lag = 19 |  |  |  |  |  |  |  |  |  |  |  |  | -0.068 | 0.030 | 0.026 |  | -0.039 | 0.069 | 0.577 |  | -0.148 | 0.070 | 0.039 |
| Lag = 20 |  |  |  |  |  |  |  |  |  |  |  |  | -0.043 | 0.024 | 0.081 |  | -0.101 | 0.050 | 0.046 |  | -0.185 | 0.070 | 0.010 |
| Lag = 21 |  |  |  |  |  |  |  |  |  |  |  |  |  |  |  |  | -0.132 | 0.049 | 0.009 |  | -0.184 | 0.069 | 0.010 |
| Lag = 22 |  |  |  |  |  |  |  |  |  |  |  |  |  |  |  |  | -0.064 | 0.049 | 0.201 |  | -0.123 | 0.069 | 0.078 |
| Lag = 23 |  |  |  |  |  |  |  |  |  |  |  |  |  |  |  |  | -0.042 | 0.041 | 0.315 |  | -0.122 | 0.061 | 0.049 |
| Lag = 24 |  |  |  |  |  |  |  |  |  |  |  |  |  |  |  |  | -0.082 | 0.029 | 0.006 |  | -0.190 | 0.042 | 0.000 |
| Lag = 25 |  |  |  |  |  |  |  |  |  |  |  |  |  |  |  |  | -0.075 | 0.024 | 0.002 |  | -0.188 | 0.041 | 0.000 |
| Lag = 26 |  |  |  |  |  |  |  |  |  |  |  |  |  |  |  |  |  |  |  |  | -0.156 | 0.041 | 0.000 |
| Lag = 27 |  |  |  |  |  |  |  |  |  |  |  |  |  |  |  |  |  |  |  |  | -0.106 | 0.035 | 0.003 |
| Lag = 28 |  |  |  |  |  |  |  |  |  |  |  |  |  |  |  |  |  |  |  |  | -0.086 | 0.025 | 0.001 |
| Lag = 29 |  |  |  |  |  |  |  |  |  |  |  |  |  |  |  |  |  |  |  |  | -0.075 | 0.020 | 0.001 |
| **Daily Additional Sick Posts** |  |  |  |  |  |  |  |  |  |  |  |  |  |  |  |  |  |  |  |  |  |  |  |
| Lag = 1 | 2.420 | 0.430 | 0.000 |  | 1.750 | 0.374 | 0.000 |  | 1.780 | 0.352 | 0.000 |  | 1.709 | 0.336 | 0.000 |  | 1.631 | 0.298 | 0.000 |  | 1.368 | 0.246 | 0.000 |
| Lag = 2 | 2.113 | 0.445 | 0.000 |  | 1.774 | 0.377 | 0.000 |  | 1.802 | 0.330 | 0.000 |  | 1.869 | 0.331 | 0.000 |  | 1.579 | 0.294 | 0.000 |  | 1.409 | 0.252 | 0.000 |
| Lag = 3 | 3.296 | 0.472 | 0.000 |  | 2.732 | 0.398 | 0.000 |  | 2.637 | 0.339 | 0.000 |  | 2.554 | 0.343 | 0.000 |  | 2.126 | 0.313 | 0.000 |  | 1.928 | 0.265 | 0.000 |
| Lag = 4 | 2.177 | 0.504 | 0.000 |  | 2.379 | 0.415 | 0.000 |  | 2.718 | 0.339 | 0.000 |  | 2.848 | 0.353 | 0.000 |  | 2.404 | 0.322 | 0.000 |  | 2.472 | 0.273 | 0.000 |
| Lag = 5 | 2.010 | 0.493 | 0.000 |  | 2.606 | 0.430 | 0.000 |  | 2.987 | 0.345 | 0.000 |  | 2.769 | 0.372 | 0.000 |  | 2.511 | 0.333 | 0.000 |  | 2.767 | 0.270 | 0.000 |
| Lag = 6 |  |  |  |  | 3.611 | 0.454 | 0.000 |  | 3.546 | 0.358 | 0.000 |  | 3.347 | 0.360 | 0.000 |  | 3.151 | 0.322 | 0.000 |  | 3.285 | 0.259 | 0.000 |
| Lag = 7 |  |  |  |  | 2.246 | 0.578 | 0.000 |  | 2.614 | 0.457 | 0.000 |  | 2.142 | 0.450 | 0.000 |  | 2.561 | 0.420 | 0.000 |  | 2.792 | 0.335 | 0.000 |
| Lag = 8 |  |  |  |  | 1.712 | 0.656 | 0.010 |  | 2.545 | 0.532 | 0.000 |  | 1.975 | 0.532 | 0.000 |  | 2.429 | 0.508 | 0.000 |  | 2.842 | 0.407 | 0.000 |
| Lag = 9 |  |  |  |  | 0.962 | 0.634 | 0.132 |  | 2.661 | 0.555 | 0.000 |  | 1.889 | 0.565 | 0.001 |  | 2.392 | 0.557 | 0.000 |  | 3.021 | 0.454 | 0.000 |
| Lag = 10 |  |  |  |  | 0.834 | 0.536 | 0.123 |  | 3.082 | 0.552 | 0.000 |  | 2.288 | 0.574 | 0.000 |  | 2.724 | 0.582 | 0.000 |  | 3.607 | 0.483 | 0.000 |
| Lag = 11 |  |  |  |  |  |  |  |  | 3.415 | 0.569 | 0.000 |  | 3.045 | 0.587 | 0.000 |  | 3.301 | 0.611 | 0.000 |  | 4.031 | 0.504 | 0.000 |
| Lag = 12 |  |  |  |  |  |  |  |  | 3.639 | 0.577 | 0.000 |  | 3.537 | 0.584 | 0.000 |  | 3.813 | 0.573 | 0.000 |  | 4.978 | 0.493 | 0.000 |
| Lag = 13 |  |  |  |  |  |  |  |  | 3.589 | 0.602 | 0.000 |  | 3.251 | 0.645 | 0.000 |  | 3.475 | 0.635 | 0.000 |  | 4.820 | 0.551 | 0.000 |
| Lag = 14 |  |  |  |  |  |  |  |  | 3.444 | 0.597 | 0.000 |  | 2.706 | 0.693 | 0.000 |  | 2.148 | 0.679 | 0.002 |  | 4.126 | 0.639 | 0.000 |
| Lag = 15 |  |  |  |  |  |  |  |  | 1.993 | 0.495 | 0.000 |  | 1.132 | 0.671 | 0.096 |  | 0.459 | 0.667 | 0.494 |  | 2.465 | 0.652 | 0.000 |
| Lag = 16 |  |  |  |  |  |  |  |  |  |  |  |  | -0.492 | 0.743 | 0.510 |  | -1.169 | 0.739 | 0.118 |  | -0.190 | 0.698 | 0.786 |
| Lag = 17 |  |  |  |  |  |  |  |  |  |  |  |  | 0.726 | 0.749 | 0.335 |  | 1.870 | 0.766 | 0.017 |  | 2.089 | 0.756 | 0.007 |
| Lag = 18 |  |  |  |  |  |  |  |  |  |  |  |  | 0.409 | 0.775 | 0.599 |  | 1.071 | 0.821 | 0.196 |  | 0.716 | 0.729 | 0.330 |
| Lag = 19 |  |  |  |  |  |  |  |  |  |  |  |  | -0.169 | 0.785 | 0.830 |  | -1.362 | 0.791 | 0.089 |  | -0.759 | 0.725 | 0.299 |
| Lag = 20 |  |  |  |  |  |  |  |  |  |  |  |  | 0.310 | 0.686 | 0.653 |  | -0.454 | 0.745 | 0.544 |  | 0.184 | 0.713 | 0.797 |
| Lag = 21 |  |  |  |  |  |  |  |  |  |  |  |  |  |  |  |  | 0.497 | 0.762 | 0.517 |  | 1.001 | 0.727 | 0.174 |
| Lag = 22 |  |  |  |  |  |  |  |  |  |  |  |  |  |  |  |  | -0.393 | 0.752 | 0.602 |  | 0.728 | 0.716 | 0.313 |
| Lag = 23 |  |  |  |  |  |  |  |  |  |  |  |  |  |  |  |  | 0.341 | 0.753 | 0.652 |  | 0.117 | 0.690 | 0.866 |
| Lag = 24 |  |  |  |  |  |  |  |  |  |  |  |  |  |  |  |  | -0.215 | 0.742 | 0.773 |  | -0.238 | 0.638 | 0.710 |
| Lag = 25 |  |  |  |  |  |  |  |  |  |  |  |  |  |  |  |  | -0.707 | 0.666 | 0.292 |  | 0.018 | 0.623 | 0.978 |
| Lag = 26 |  |  |  |  |  |  |  |  |  |  |  |  |  |  |  |  |  |  |  |  | 0.603 | 0.618 | 0.333 |
| Lag = 27 |  |  |  |  |  |  |  |  |  |  |  |  |  |  |  |  |  |  |  |  | 0.193 | 0.632 | 0.761 |
| Lag = 28 |  |  |  |  |  |  |  |  |  |  |  |  |  |  |  |  |  |  |  |  | -0.036 | 0.635 | 0.955 |
| Lag = 29 |  |  |  |  |  |  |  |  |  |  |  |  |  |  |  |  |  |  |  |  | 0.076 | 0.556 | 0.891 |
|  |  | | |  |  | | |  |  | | |  |  | | |  |  | | |  |  | | |
| Adjusted R^2^ | 0.900 | | |  | 0.936 | | |  | 0.964 | | |  | 0.970 | | |  | 0.978 | | |  | 0.986 | | |

## Table E. Model summaries for other COVID-19 posts (difference scores) predicting new cases (difference scores) in mainland China with varying lag terms (*N* = 122)

|  | **Model 1 (max lag = 5)** | | |  | **Model 2 (max lag = 10)** | | |  | **Model 3 (max lag = 15)** | | |  | **Model 4 (max lag = 20)** | | |  | **Model 5 (max lag = 25)** | | |  | **Model 6 (max lag = 29)** | | |
| --- | --- | --- | --- | --- | --- | --- | --- | --- | --- | --- | --- | --- | --- | --- | --- | --- | --- | --- | --- | --- | --- | --- | --- |
|  | ***B*** | ***SE*** | ***p*** |  | ***B*** | ***SE*** | ***p*** |  | ***B*** | ***SE*** | ***p*** |  | ***B*** | ***SE*** | ***p*** |  | ***B*** | ***SE*** | ***p*** |  | ***B*** | ***SE*** | ***p*** |
| Intercept | -107.415 | 54.809 | 0.053 |  | -112.068 | 51.926 | 0.033 |  | -118.982 | 41.204 | 0.005 |  | -115.328 | 30.459 | 0.000 |  | -109.323 | 18.228 | 0.000 |  | -101.757 | 16.492 | 0.000 |
| Change of Diagnosis Criteria | 12843.360 | 621.189 | 0.000 |  | 12869.610 | 616.865 | 0.000 |  | 12845.750 | 528.905 | 0.000 |  | 11941.980 | 747.882 | 0.000 |  | 12852.910 | 695.133 | 0.000 |  | 11878.000 | 810.914 | 0.000 |
| **Daily-Additional New Cases** |  |  |  |  |  |  |  |  |  |  |  |  |  |  |  |  |  |  |  |  |  |  |  |
| Lag = 1 | -0.585 | 0.043 | 0.000 |  | -0.585 | 0.043 | 0.000 |  | -0.639 | 0.037 | 0.000 |  | -0.563 | 0.049 | 0.000 |  | -0.535 | 0.046 | 0.000 |  | -0.673 | 0.060 | 0.000 |
| Lag = 2 | -0.451 | 0.050 | 0.000 |  | -0.472 | 0.051 | 0.000 |  | -0.546 | 0.045 | 0.000 |  | -0.529 | 0.043 | 0.000 |  | -0.307 | 0.055 | 0.000 |  | -0.530 | 0.083 | 0.000 |
| Lag = 3 | -0.320 | 0.053 | 0.000 |  | -0.371 | 0.055 | 0.000 |  | -0.492 | 0.049 | 0.000 |  | -0.511 | 0.038 | 0.000 |  | -0.172 | 0.064 | 0.009 |  | -0.436 | 0.100 | 0.000 |
| Lag = 4 | -0.188 | 0.050 | 0.000 |  | -0.268 | 0.057 | 0.000 |  | -0.402 | 0.050 | 0.000 |  | -0.354 | 0.045 | 0.000 |  | 0.030 | 0.067 | 0.655 |  | -0.241 | 0.116 | 0.042 |
| Lag = 5 | -0.082 | 0.043 | 0.059 |  | -0.155 | 0.057 | 0.008 |  | -0.300 | 0.050 | 0.000 |  | -0.324 | 0.040 | 0.000 |  | 0.067 | 0.060 | 0.263 |  | -0.088 | 0.102 | 0.394 |
| Lag = 6 |  |  |  |  | -0.078 | 0.057 | 0.174 |  | -0.205 | 0.049 | 0.000 |  | -0.281 | 0.039 | 0.000 |  | 0.034 | 0.053 | 0.521 |  | 0.008 | 0.077 | 0.922 |
| Lag = 7 |  |  |  |  | -0.092 | 0.056 | 0.105 |  | -0.230 | 0.050 | 0.000 |  | -0.310 | 0.042 | 0.000 |  | -0.002 | 0.050 | 0.976 |  | -0.003 | 0.067 | 0.958 |
| Lag = 8 |  |  |  |  | -0.055 | 0.055 | 0.319 |  | -0.209 | 0.050 | 0.000 |  | -0.289 | 0.042 | 0.000 |  | -0.091 | 0.040 | 0.024 |  | -0.036 | 0.069 | 0.606 |
| Lag = 9 |  |  |  |  | -0.063 | 0.050 | 0.207 |  | -0.235 | 0.049 | 0.000 |  | -0.315 | 0.041 | 0.000 |  | -0.187 | 0.032 | 0.000 |  | -0.081 | 0.064 | 0.209 |
| Lag = 10 |  |  |  |  | -0.035 | 0.042 | 0.404 |  | -0.200 | 0.047 | 0.000 |  | -0.300 | 0.039 | 0.000 |  | -0.136 | 0.033 | 0.000 |  | -0.104 | 0.053 | 0.052 |
| Lag = 11 |  |  |  |  |  |  |  |  | -0.192 | 0.046 | 0.000 |  | -0.293 | 0.038 | 0.000 |  | -0.132 | 0.035 | 0.000 |  | -0.154 | 0.055 | 0.007 |
| Lag = 12 |  |  |  |  |  |  |  |  | -0.194 | 0.045 | 0.000 |  | -0.311 | 0.037 | 0.000 |  | -0.099 | 0.038 | 0.012 |  | -0.154 | 0.043 | 0.001 |
| Lag = 13 |  |  |  |  |  |  |  |  | -0.162 | 0.044 | 0.000 |  | -0.256 | 0.041 | 0.000 |  | -0.008 | 0.039 | 0.835 |  | -0.084 | 0.046 | 0.071 |
| Lag = 14 |  |  |  |  |  |  |  |  | -0.128 | 0.040 | 0.002 |  | -0.264 | 0.037 | 0.000 |  | -0.010 | 0.037 | 0.786 |  | -0.059 | 0.038 | 0.123 |
| Lag = 15 |  |  |  |  |  |  |  |  | -0.092 | 0.034 | 0.008 |  | -0.252 | 0.034 | 0.000 |  | -0.056 | 0.034 | 0.110 |  | -0.064 | 0.037 | 0.093 |
| Lag = 16 |  |  |  |  |  |  |  |  |  |  |  |  | -0.191 | 0.037 | 0.000 |  | -0.029 | 0.033 | 0.382 |  | -0.015 | 0.038 | 0.689 |
| Lag = 17 |  |  |  |  |  |  |  |  |  |  |  |  | -0.178 | 0.034 | 0.000 |  | -0.012 | 0.032 | 0.716 |  | 0.008 | 0.037 | 0.841 |
| Lag = 18 |  |  |  |  |  |  |  |  |  |  |  |  | -0.170 | 0.032 | 0.000 |  | -0.035 | 0.032 | 0.273 |  | -0.028 | 0.036 | 0.448 |
| Lag = 19 |  |  |  |  |  |  |  |  |  |  |  |  | -0.120 | 0.030 | 0.000 |  | -0.039 | 0.031 | 0.210 |  | -0.045 | 0.032 | 0.166 |
| Lag = 20 |  |  |  |  |  |  |  |  |  |  |  |  | -0.086 | 0.025 | 0.001 |  | -0.060 | 0.029 | 0.045 |  | -0.059 | 0.031 | 0.059 |
| Lag = 21 |  |  |  |  |  |  |  |  |  |  |  |  |  |  |  |  | -0.021 | 0.034 | 0.537 |  | -0.044 | 0.032 | 0.174 |
| Lag = 22 |  |  |  |  |  |  |  |  |  |  |  |  |  |  |  |  | 0.064 | 0.032 | 0.051 |  | -0.008 | 0.033 | 0.814 |
| Lag = 23 |  |  |  |  |  |  |  |  |  |  |  |  |  |  |  |  | 0.060 | 0.029 | 0.038 |  | -0.005 | 0.032 | 0.883 |
| Lag = 24 |  |  |  |  |  |  |  |  |  |  |  |  |  |  |  |  | 0.050 | 0.024 | 0.041 |  | 0.012 | 0.033 | 0.716 |
| Lag = 25 |  |  |  |  |  |  |  |  |  |  |  |  |  |  |  |  | 0.031 | 0.019 | 0.104 |  | 0.019 | 0.034 | 0.581 |
| Lag = 26 |  |  |  |  |  |  |  |  |  |  |  |  |  |  |  |  |  |  |  |  | 0.001 | 0.032 | 0.974 |
| Lag = 27 |  |  |  |  |  |  |  |  |  |  |  |  |  |  |  |  |  |  |  |  | -0.009 | 0.032 | 0.784 |
| Lag = 28 |  |  |  |  |  |  |  |  |  |  |  |  |  |  |  |  |  |  |  |  | -0.023 | 0.027 | 0.381 |
| Lag = 29 |  |  |  |  |  |  |  |  |  |  |  |  |  |  |  |  |  |  |  |  | -0.004 | 0.021 | 0.858 |
| **Daily-Additional Other COVID-19 Posts** | |  |  |  |  |  |  |  |  |  |  |  |  |  |  |  |  |  |  |  |  |  |  |
| Lag = 1 | 0.031 | 0.030 | 0.031 |  | 0.029 | 0.029 | 0.335 |  | 0.033 | 0.025 | 0.185 |  | 0.034 | 0.019 | 0.080 |  | 0.018 | 0.011 | 0.117 |  | 0.016 | 0.010 | 0.117 |
| Lag = 2 | 0.044 | 0.030 | 0.044 |  | 0.060 | 0.029 | 0.044 |  | 0.069 | 0.025 | 0.007 |  | 0.046 | 0.019 | 0.019 |  | 0.043 | 0.011 | 0.000 |  | 0.039 | 0.010 | 0.000 |
| Lag = 3 | 0.040 | 0.029 | 0.040 |  | 0.032 | 0.029 | 0.288 |  | 0.015 | 0.024 | 0.525 |  | 0.006 | 0.019 | 0.761 |  | -0.007 | 0.012 | 0.562 |  | -0.005 | 0.010 | 0.642 |
| Lag = 4 | 0.044 | 0.031 | 0.044 |  | 0.050 | 0.032 | 0.119 |  | 0.040 | 0.026 | 0.121 |  | 0.043 | 0.022 | 0.052 |  | 0.041 | 0.013 | 0.003 |  | 0.039 | 0.011 | 0.001 |
| Lag = 5 | 0.040 | 0.031 | 0.040 |  | 0.038 | 0.031 | 0.230 |  | 0.039 | 0.026 | 0.144 |  | 0.062 | 0.022 | 0.005 |  | 0.038 | 0.013 | 0.005 |  | 0.040 | 0.012 | 0.001 |
| Lag = 6 |  |  |  |  | 0.066 | 0.032 | 0.042 |  | 0.076 | 0.026 | 0.005 |  | 0.074 | 0.022 | 0.001 |  | 0.063 | 0.013 | 0.000 |  | 0.069 | 0.012 | 0.000 |
| Lag = 7 |  |  |  |  | 0.022 | 0.032 | 0.500 |  | 0.058 | 0.027 | 0.035 |  | 0.053 | 0.022 | 0.020 |  | 0.031 | 0.014 | 0.033 |  | 0.046 | 0.013 | 0.001 |
| Lag = 8 |  |  |  |  | 0.036 | 0.030 | 0.238 |  | 0.043 | 0.027 | 0.119 |  | 0.022 | 0.022 | 0.325 |  | 0.010 | 0.014 | 0.490 |  | 0.031 | 0.014 | 0.031 |
| Lag = 9 |  |  |  |  | 0.050 | 0.031 | 0.116 |  | 0.065 | 0.029 | 0.027 |  | 0.060 | 0.022 | 0.009 |  | 0.024 | 0.014 | 0.098 |  | 0.039 | 0.014 | 0.006 |
| Lag = 10 |  |  |  |  | 0.094 | 0.031 | 0.003 |  | 0.090 | 0.028 | 0.002 |  | 0.088 | 0.022 | 0.000 |  | 0.051 | 0.014 | 0.001 |  | 0.053 | 0.013 | 0.000 |
| Lag = 11 |  |  |  |  |  |  |  |  | 0.064 | 0.029 | 0.030 |  | 0.067 | 0.022 | 0.003 |  | 0.034 | 0.014 | 0.020 |  | 0.039 | 0.013 | 0.005 |
| Lag = 12 |  |  |  |  |  |  |  |  | 0.052 | 0.029 | 0.071 |  | 0.085 | 0.022 | 0.000 |  | 0.043 | 0.014 | 0.003 |  | 0.057 | 0.013 | 0.000 |
| Lag = 13 |  |  |  |  |  |  |  |  | 0.077 | 0.028 | 0.007 |  | 0.099 | 0.023 | 0.000 |  | 0.078 | 0.014 | 0.000 |  | 0.096 | 0.013 | 0.000 |
| Lag = 14 |  |  |  |  |  |  |  |  | 0.091 | 0.029 | 0.002 |  | 0.094 | 0.024 | 0.000 |  | 0.046 | 0.016 | 0.006 |  | 0.066 | 0.016 | 0.000 |
| Lag = 15 |  |  |  |  |  |  |  |  | 0.122 | 0.028 | 0.000 |  | 0.085 | 0.024 | 0.001 |  | 0.053 | 0.017 | 0.003 |  | 0.070 | 0.017 | 0.000 |
| Lag = 16 |  |  |  |  |  |  |  |  |  |  |  |  | 0.005 | 0.025 | 0.851 |  | -0.059 | 0.017 | 0.001 |  | -0.043 | 0.017 | 0.017 |
| Lag = 17 |  |  |  |  |  |  |  |  |  |  |  |  | 0.081 | 0.025 | 0.002 |  | 0.014 | 0.017 | 0.403 |  | 0.021 | 0.017 | 0.216 |
| Lag = 18 |  |  |  |  |  |  |  |  |  |  |  |  | 0.107 | 0.028 | 0.000 |  | 0.039 | 0.020 | 0.061 |  | 0.049 | 0.021 | 0.021 |
| Lag = 19 |  |  |  |  |  |  |  |  |  |  |  |  | 0.000 | 0.042 | 0.995 |  | -0.006 | 0.030 | 0.842 |  | -0.024 | 0.038 | 0.520 |
| Lag = 20 |  |  |  |  |  |  |  |  |  |  |  |  | 0.111 | 0.042 | 0.009 |  | 0.053 | 0.028 | 0.068 |  | 0.021 | 0.035 | 0.547 |
| Lag = 21 |  |  |  |  |  |  |  |  |  |  |  |  |  |  |  |  | 0.024 | 0.033 | 0.467 |  | 0.036 | 0.038 | 0.349 |
| Lag = 22 |  |  |  |  |  |  |  |  |  |  |  |  |  |  |  |  | -0.068 | 0.037 | 0.072 |  | 0.004 | 0.039 | 0.927 |
| Lag = 23 |  |  |  |  |  |  |  |  |  |  |  |  |  |  |  |  | -0.116 | 0.036 | 0.002 |  | -0.051 | 0.038 | 0.187 |
| Lag = 24 |  |  |  |  |  |  |  |  |  |  |  |  |  |  |  |  | -0.185 | 0.035 | 0.000 |  | -0.103 | 0.042 | 0.017 |
| Lag = 25 |  |  |  |  |  |  |  |  |  |  |  |  |  |  |  |  | -0.156 | 0.035 | 0.000 |  | -0.105 | 0.035 | 0.003 |
| Lag = 26 |  |  |  |  |  |  |  |  |  |  |  |  |  |  |  |  |  |  |  |  | -0.070 | 0.033 | 0.039 |
| Lag = 27 |  |  |  |  |  |  |  |  |  |  |  |  |  |  |  |  |  |  |  |  | -0.101 | 0.041 | 0.016 |
| Lag = 28 |  |  |  |  |  |  |  |  |  |  |  |  |  |  |  |  |  |  |  |  | -0.052 | 0.053 | 0.327 |
| Lag = 29 |  |  |  |  |  |  |  |  |  |  |  |  |  |  |  |  |  |  |  |  | -0.026 | 0.053 | 0.629 |
|  |  | | |  |  | | |  |  | | |  |  | | |  |  | | |  |  | | |
| Adjusted R^2^ | 0.846 | | |  | 0.862 | | |  | 0.913 | | |  | 0.954 | | |  | 0.984 | | |  | 0.988 | | |

## Table F. Model comparisons for sick posts or other COVID-19 posts (difference scores) predicting new cases (difference scores) in mainland China with varying linear decay rates of the effect of the changed diagnostic criteria on February 12^th^, 2020 (*N* = 122)

Hubei province adopted the fifth edition of the diagnostic criteria on Feb. 12th, 2020. We compared models with different decay functions of this change’s intervention effects, including an “instant pulse” on Feb. 12th (the indicator was coded as 1 at February 12 and 0 elsewhere), and linear decays in 2 days (the indicator was coded as 1 and 0.5 at February 12^th^ and 13^th^), 3 days (the indicator was coded as 1, 0.667, and 0.333 at February 12^th^, 13^th^, and 14^th^), 4 days (the indicator was coded as 1, 0.75, 0.5, and 0.25 from February 12^th^ to 15^th^), or days 5 days (the indicator variable was coded as 1, 0.8, 0.6, 0.4, .2 from February 12^th^ to 16^th^).

|  | **Cases Regressed on Sick Posts** | | | | |  | **Cases Regressed on Other COVID-19 Posts** | | | | |
| --- | --- | --- | --- | --- | --- | --- | --- | --- | --- | --- | --- |
|  | **Adjusted R^2^** | **AIC** | **BIC** | **Model df** | **Residual df** |  | **Adjusted R^2^** | **AIC** | **BIC** | **Model df** | **Residual df** |
| Instant Pulse | 0.970 | 1741.721 | 1859.490 | 41 | 80 |  | 0.954 | 1792.342 | 1910.110 | 41 | 80 |
| 2 days | 0.945 | 1815.086 | 1932.855 | 41 | 80 |  | 0.913 | 1871.580 | 1989.349 | 41 | 80 |
| 3 days | 0.924 | 1854.804 | 1972.573 | 41 | 80 |  | 0.876 | 1914.615 | 2032.384 | 41 | 80 |
| 4 days | 0.906 | 1881.009 | 1998.778 | 41 | 80 |  | 0.849 | 1938.742 | 2056.511 | 41 | 80 |
| 5 days | 0.892 | 1897.689 | 2015.458 | 41 | 80 |  | 0.832 | 1951.363 | 2069.131 | 41 | 80 |

## Table G. Model summaries of sick posts or other COVID-19 posts (difference scores) predicting new cases (difference scores) in mainland China, including a baseline model without effects of social media posts (*N* = 122)

|  | **Model 1 (Baseline)** | | |  | **Model 2 (Sick Posts)** | | |  | **Model 3 (Other COVID-19 Posts)** | | |
| --- | --- | --- | --- | --- | --- | --- | --- | --- | --- | --- | --- |
|  | ***B*** | ***SE*** | ***p*** |  | ***B*** | ***SE*** | ***p*** |  | ***B*** | ***SE*** | ***p*** |
|  |  |  |  |  |  |  |  |  |  |  |  |
| Intercept | -106.565 | 55.625 | 0.058 |  | -99.941 | 22.958 | 0.000 |  | -115.328 | 28.784 | 0.000 |
| Change of Diagnosis Criteria | 13152.220 | 223.193 | 0.000 |  | 11476.130 | 682.503 | 0.000 |  | 11941.980 | 678.476 | 0.000 |
| **Daily Additional New Cases** |  |  |  |  |  |  |  |  |  |  |  |
| Lag = 1 | -0.568 | 0.175 | 0.002 |  | -0.686 | 0.072 | 0.000 |  | -0.563 | 0.068 | 0.000 |
| Lag = 2 | -0.424 | 0.179 | 0.020 |  | -0.569 | 0.090 | 0.000 |  | -0.529 | 0.068 | 0.000 |
| Lag = 3 | -0.302 | 0.162 | 0.066 |  | -0.582 | 0.082 | 0.000 |  | -0.511 | 0.069 | 0.000 |
| Lag = 4 | -0.182 | 0.136 | 0.183 |  | -0.524 | 0.079 | 0.000 |  | -0.354 | 0.065 | 0.000 |
| Lag = 5 | -0.095 | 0.113 | 0.401 |  | -0.544 | 0.082 | 0.000 |  | -0.324 | 0.062 | 0.000 |
| Lag = 6 | -0.031 | 0.088 | 0.728 |  | -0.511 | 0.102 | 0.000 |  | -0.281 | 0.058 | 0.000 |
| Lag = 7 | -0.049 | 0.068 | 0.476 |  | -0.465 | 0.112 | 0.000 |  | -0.310 | 0.055 | 0.000 |
| Lag = 8 | -0.034 | 0.055 | 0.540 |  | -0.429 | 0.101 | 0.000 |  | -0.289 | 0.054 | 0.000 |
| Lag = 9 | -0.082 | 0.045 | 0.070 |  | -0.377 | 0.099 | 0.000 |  | -0.315 | 0.053 | 0.000 |
| Lag = 10 | -0.093 | 0.044 | 0.036 |  | -0.284 | 0.082 | 0.001 |  | -0.300 | 0.050 | 0.000 |
| Lag = 11 | -0.129 | 0.048 | 0.009 |  | -0.317 | 0.081 | 0.000 |  | -0.293 | 0.048 | 0.000 |
| Lag = 12 | -0.148 | 0.057 | 0.011 |  | -0.275 | 0.078 | 0.001 |  | -0.311 | 0.049 | 0.000 |
| Lag = 13 | -0.167 | 0.066 | 0.014 |  | -0.169 | 0.079 | 0.036 |  | -0.256 | 0.055 | 0.000 |
| Lag = 14 | -0.176 | 0.075 | 0.021 |  | -0.137 | 0.070 | 0.054 |  | -0.264 | 0.058 | 0.000 |
| Lag = 15 | -0.182 | 0.081 | 0.028 |  | -0.143 | 0.055 | 0.011 |  | -0.252 | 0.056 | 0.000 |
| Lag = 16 | -0.168 | 0.089 | 0.060 |  | -0.121 | 0.058 | 0.039 |  | -0.191 | 0.063 | 0.003 |
| Lag = 17 | -0.141 | 0.093 | 0.136 |  | -0.125 | 0.055 | 0.026 |  | -0.178 | 0.066 | 0.009 |
| Lag = 18 | -0.139 | 0.098 | 0.161 |  | -0.113 | 0.053 | 0.037 |  | -0.170 | 0.070 | 0.017 |
| Lag = 19 | -0.109 | 0.099 | 0.271 |  | -0.068 | 0.048 | 0.159 |  | -0.120 | 0.074 | 0.106 |
| Lag = 20 | -0.069 | 0.093 | 0.461 |  | -0.043 | 0.046 | 0.354 |  | -0.086 | 0.072 | 0.233 |
| **Daily Additional Posts** |  |  |  |  |  |  |  |  |  |  |  |
| Lag = 1 | – | – | – |  | 1.709 | 0.441 | 0.000 |  | 0.034 | 0.017 | 0.056 |
| Lag = 2 | – | – | – |  | 1.869 | 0.428 | 0.000 |  | 0.046 | 0.024 | 0.060 |
| Lag = 3 | – | – | – |  | 2.554 | 0.531 | 0.000 |  | 0.006 | 0.021 | 0.775 |
| Lag = 4 | – | – | – |  | 2.848 | 0.465 | 0.000 |  | 0.043 | 0.017 | 0.012 |
| Lag = 5 | – | – | – |  | 2.769 | 0.443 | 0.000 |  | 0.062 | 0.025 | 0.014 |
| Lag = 6 | – | – | – |  | 3.347 | 0.481 | 0.000 |  | 0.074 | 0.018 | 0.000 |
| Lag = 7 | – | – | – |  | 2.142 | 0.642 | 0.001 |  | 0.053 | 0.018 | 0.004 |
| Lag = 8 | – | – | – |  | 1.975 | 0.687 | 0.005 |  | 0.022 | 0.019 | 0.236 |
| Lag = 9 | – | – | – |  | 1.889 | 0.723 | 0.011 |  | 0.060 | 0.016 | 0.000 |
| Lag = 10 | – | – | – |  | 2.288 | 0.695 | 0.001 |  | 0.088 | 0.017 | 0.000 |
| Lag = 11 | – | – | – |  | 3.045 | 0.793 | 0.000 |  | 0.067 | 0.019 | 0.001 |
| Lag = 12 | – | – | – |  | 3.537 | 0.914 | 0.000 |  | 0.085 | 0.016 | 0.000 |
| Lag = 13 | – | – | – |  | 3.251 | 0.943 | 0.001 |  | 0.099 | 0.018 | 0.000 |
| Lag = 14 | – | – | – |  | 2.706 | 0.889 | 0.003 |  | 0.094 | 0.019 | 0.000 |
| Lag = 15 | – | – | – |  | 1.132 | 1.007 | 0.264 |  | 0.085 | 0.026 | 0.002 |
| Lag = 16 | – | – | – |  | -0.492 | 0.961 | 0.610 |  | 0.005 | 0.024 | 0.842 |
| Lag = 17 | – | – | – |  | 0.726 | 1.017 | 0.477 |  | 0.081 | 0.020 | 0.000 |
| Lag = 18 | – | – | – |  | 0.409 | 0.935 | 0.663 |  | 0.107 | 0.031 | 0.001 |
| Lag = 19 | – | – | – |  | -0.169 | 0.984 | 0.864 |  | 0.000 | 0.042 | 0.995 |
| Lag = 20 | – | – | – |  | 0.310 | 0.800 | 0.700 |  | 0.111 | 0.046 | 0.017 |
|  |  | | |  |  | | |  |  | | |
| R^2^ (Δ) | 0.869 | | |  | 0.980 (0.111) | | |  | 0.970 (0.101) | | |
| Adjusted-R^2^ (Δ) | 0.842 | | |  | 0.970 (0.128) | | |  | 0.954 (0.112) | | |
| AIC | 1931.403 | | |  | 1741.721 | | |  | 1792.342 | | |
| BIC | 1993.092 | | |  | 1859.490 | | |  | 1910.110 | | |

Note. Robust standard errors are reported. ΔR^2^ is compared with the baseline model.

## Table H. Model summaries of sick posts or other COVID-19 posts (difference scores) predicting new cases (difference scores) within or outside Hubei (*N* = 122)

|  | **Cases Regressed on Sick Posts** | | | | | | |  | **Cases Regressed on Other COVID-19 Posts** | | | | | | |
| --- | --- | --- | --- | --- | --- | --- | --- | --- | --- | --- | --- | --- | --- | --- | --- |
|  | **Model 1****(Hubei)** | | |  | **Model 2** **(outside Hubei)** | | |  | **Model 3** **(Hubei)** | | |  | **Model 4** **(Outside Hubei)** | | |
|  | ***B*** | ***SE*** | ***p*** |  | ***B*** | ***SE*** | ***p*** |  | ***B*** | ***SE*** | ***p*** |  | ***B*** | ***SE*** | ***p*** |
| Intercept | -11.733 | 36.274 | 0.747 |  | -1.033 | 2.257 | 0.648 |  | 30.142 | 50.933 | 0.556 |  | -1.535 | 1.916 | 0.425 |
| Change of Diagnosis Criteria | 13433.280 | 263.711 | 0.000 |  | -158.715 | 109.008 | 0.149 |  | 13316.680 | 299.307 | 0.000 |  | 126.976 | 84.275 | 0.136 |
| **Daily Additional New Cases** |  |  |  |  |  |  |  |  |  |  |  |  |  |  |  |
| Lag = 1 | -0.654 | 0.074 | 0.000 |  | -0.197 | 0.154 | 0.204 |  | -0.580 | 0.135 | 0.000 |  | -0.734 | 0.211 | 0.001 |
| Lag = 2 | -0.577 | 0.087 | 0.000 |  | -0.379 | 0.191 | 0.051 |  | -0.475 | 0.145 | 0.002 |  | -0.331 | 0.190 | 0.084 |
| Lag = 3 | -0.506 | 0.088 | 0.000 |  | -0.330 | 0.142 | 0.023 |  | -0.361 | 0.137 | 0.010 |  | -0.316 | 0.169 | 0.066 |
| Lag = 4 | -0.447 | 0.085 | 0.000 |  | -0.091 | 0.225 | 0.686 |  | -0.269 | 0.123 | 0.032 |  | -0.111 | 0.208 | 0.594 |
| Lag = 5 | -0.408 | 0.084 | 0.000 |  | -0.245 | 0.184 | 0.188 |  | -0.201 | 0.110 | 0.072 |  | 0.264 | 0.230 | 0.254 |
| Lag = 6 | -0.364 | 0.078 | 0.000 |  | 0.027 | 0.180 | 0.883 |  | -0.147 | 0.095 | 0.127 |  | 0.551 | 0.210 | 0.011 |
| Lag = 7 | -0.391 | 0.076 | 0.000 |  | -0.002 | 0.163 | 0.992 |  | -0.179 | 0.083 | 0.034 |  | 0.367 | 0.177 | 0.042 |
| Lag = 8 | -0.385 | 0.070 | 0.000 |  | -0.213 | 0.160 | 0.188 |  | -0.181 | 0.074 | 0.016 |  | 0.129 | 0.155 | 0.407 |
| Lag = 9 | -0.398 | 0.067 | 0.000 |  | -0.087 | 0.152 | 0.570 |  | -0.208 | 0.068 | 0.003 |  | 0.053 | 0.125 | 0.672 |
| Lag = 10 | -0.376 | 0.067 | 0.000 |  | -0.060 | 0.137 | 0.659 |  | -0.212 | 0.069 | 0.003 |  | -0.212 | 0.155 | 0.174 |
| Lag = 11 | -0.360 | 0.069 | 0.000 |  | -0.036 | 0.107 | 0.740 |  | -0.230 | 0.072 | 0.002 |  | -0.325 | 0.174 | 0.065 |
| Lag = 12 | -0.340 | 0.070 | 0.000 |  | -0.238 | 0.118 | 0.048 |  | -0.238 | 0.078 | 0.003 |  | -0.283 | 0.128 | 0.030 |
| Lag = 13 | -0.315 | 0.071 | 0.000 |  | -0.213 | 0.112 | 0.061 |  | -0.242 | 0.083 | 0.005 |  | -0.311 | 0.132 | 0.021 |
| Lag = 14 | -0.293 | 0.072 | 0.000 |  | -0.015 | 0.167 | 0.931 |  | -0.233 | 0.087 | 0.009 |  | -0.178 | 0.106 | 0.095 |
| Lag = 15 | -0.257 | 0.071 | 0.000 |  | -0.243 | 0.136 | 0.077 |  | -0.229 | 0.090 | 0.012 |  | -0.028 | 0.087 | 0.748 |
| Lag = 16 | -0.212 | 0.073 | 0.005 |  | -0.178 | 0.124 | 0.153 |  | -0.201 | 0.093 | 0.034 |  | -0.071 | 0.108 | 0.510 |
| Lag = 17 | -0.166 | 0.076 | 0.032 |  | -0.159 | 0.109 | 0.149 |  | -0.158 | 0.096 | 0.101 |  | 0.197 | 0.177 | 0.269 |
| Lag = 18 | -0.154 | 0.078 | 0.052 |  | -0.137 | 0.091 | 0.137 |  | -0.151 | 0.099 | 0.129 |  | 0.078 | 0.150 | 0.604 |
| Lag = 19 | -0.125 | 0.080 | 0.122 |  | -0.056 | 0.094 | 0.551 |  | -0.113 | 0.098 | 0.249 |  | -0.032 | 0.118 | 0.786 |
| Lag = 20 | -0.073 | 0.078 | 0.350 |  | -0.111 | 0.103 | 0.286 |  | -0.068 | 0.091 | 0.457 |  | -0.030 | 0.091 | 0.738 |
| **Daily Additional Posts** |  |  |  |  |  |  |  |  |  |  |  |  |  |  |  |
| Lag = 1 | 124.065 | 78.630 | 0.119 |  | 2.649 | 2.489 | 0.290 |  | 2.915 | 2.722 | 0.287 |  | 0.155 | 0.045 | 0.001 |
| Lag = 2 | 296.160 | 89.490 | 0.001 |  | 9.054 | 2.223 | 0.000 |  | 6.877 | 3.800 | 0.074 |  | 0.517 | 0.072 | 0.000 |
| Lag = 3 | 359.111 | 108.739 | 0.001 |  | 6.432 | 2.405 | 0.009 |  | 5.523 | 3.684 | 0.138 |  | 0.684 | 0.123 | 0.000 |
| Lag = 4 | 353.444 | 95.466 | 0.000 |  | 11.500 | 2.657 | 0.000 |  | 7.933 | 5.480 | 0.152 |  | 0.505 | 0.156 | 0.002 |
| Lag = 5 | 365.335 | 96.879 | 0.000 |  | 14.797 | 3.087 | 0.000 |  | 8.131 | 5.040 | 0.111 |  | 0.419 | 0.157 | 0.009 |
| Lag = 6 | 369.693 | 95.967 | 0.000 |  | 14.567 | 3.256 | 0.000 |  | 5.698 | 4.959 | 0.254 |  | 0.367 | 0.172 | 0.036 |
| Lag = 7 | 375.696 | 101.524 | 0.000 |  | 11.298 | 3.035 | 0.000 |  | 6.754 | 5.029 | 0.183 |  | 0.344 | 0.198 | 0.086 |
| Lag = 8 | 355.893 | 84.811 | 0.000 |  | 8.334 | 3.113 | 0.009 |  | 7.676 | 4.833 | 0.116 |  | 0.506 | 0.189 | 0.009 |
| Lag = 9 | 370.001 | 80.425 | 0.000 |  | 4.951 | 2.527 | 0.054 |  | 8.199 | 5.273 | 0.124 |  | 0.267 | 0.210 | 0.207 |
| Lag = 10 | 470.718 | 104.710 | 0.000 |  | 0.691 | 2.334 | 0.768 |  | 10.591 | 5.241 | 0.047 |  | -0.425 | 0.211 | 0.047 |
| Lag = 11 | 517.452 | 105.888 | 0.000 |  | 2.600 | 1.981 | 0.193 |  | 11.139 | 5.320 | 0.039 |  | -0.068 | 0.232 | 0.772 |
| Lag = 12 | 500.858 | 116.940 | 0.000 |  | -2.723 | 2.333 | 0.247 |  | 13.217 | 6.037 | 0.031 |  | 0.080 | 0.208 | 0.702 |
| Lag = 13 | 494.104 | 126.973 | 0.000 |  | 1.487 | 1.767 | 0.403 |  | 14.840 | 5.959 | 0.015 |  | -0.020 | 0.141 | 0.888 |
| Lag = 14 | 468.563 | 127.484 | 0.000 |  | 0.074 | 1.839 | 0.968 |  | 13.959 | 5.746 | 0.017 |  | 0.176 | 0.110 | 0.113 |
| Lag = 15 | 475.006 | 118.430 | 0.000 |  | -2.486 | 2.126 | 0.246 |  | 15.626 | 5.737 | 0.008 |  | 0.129 | 0.206 | 0.535 |
| Lag = 16 | 391.729 | 114.233 | 0.001 |  | -1.549 | 1.616 | 0.341 |  | 11.981 | 5.475 | 0.032 |  | 0.041 | 0.192 | 0.832 |
| Lag = 17 | 338.517 | 112.834 | 0.004 |  | -3.179 | 1.944 | 0.106 |  | 13.602 | 5.699 | 0.019 |  | -0.412 | 0.135 | 0.003 |
| Lag = 18 | 270.288 | 97.379 | 0.007 |  | -3.435 | 1.881 | 0.072 |  | 10.566 | 5.094 | 0.041 |  | -0.556 | 0.169 | 0.001 |
| Lag = 19 | 190.939 | 80.451 | 0.020 |  | 0.726 | 1.716 | 0.674 |  | 5.955 | 4.644 | 0.203 |  | 0.094 | 0.212 | 0.658 |
| Lag = 20 | 105.740 | 61.384 | 0.089 |  | -4.517 | 2.685 | 0.096 |  | 7.220 | 4.635 | 0.123 |  | 0.049 | 0.170 | 0.771 |
| R^2^ (Adjusted R^2^) | 0.952 (0.928) | | |  | 0.779 (0.666) | | |  | 0.911 (0.866) | | |  | 0.862 (0.790) | | |
| AIC | 1848.179 | | |  | 1191.557 | | |  | 1924.113 | | |  | 1134.491 | | |
| BIC | 1965.948 | | |  | 1309.326 | | |  | 2041.882 | | |  | 1252.259 | | |

Note. Robust standard errors are reported. ΔR^2^ is compared with the baseline model.
